# DLST-driven metabolic alterations link skin to blood glucose: A translational study from mouse to human

**DOI:** 10.1016/j.molmet.2026.102439

**Published:** 2026-09-28

**Authors:** Nsrein Ali, Syeda Tayyiba Rahat, Diana Motei, Mira Mäkelä, Subodh Sharma, Mikko Tulppo, Juhani Juntilla, J. Kalervo Hiltunen, Anni I. Nieminen, Steffen Ohlmeier, Seppo Vainio

**Affiliations:** 1 Faculty of Biochemistry and Molecular Medicine, University of Oulu, Oulu, 90220, Finland; 2 Infotech, University of Oulu, Oulu, 90220, Finland; 3 GeneCellNano Flagship, University of Oulu, Oulu, 90220, Finland; 4 Faculty of Medicine, University of Oulu, Oulu, 90220, Finland; 5 Medical Research Center Oulu, Cardiology, Faculty of Medicine, University of Oulu, Finland; 6 Resaerch Unit of Biomedicine and Internal Medicine, Faculty of Medicine, University of Oulu, Finland; 7 Stem Cell and Metabolism Research Program, Faculty of Medicine, University of Helsinki, Finland; 8 Proteomics and Protein Analysis Core Facility, Biocenter Oulu, University of Oulu, Oulu, Finland; 9 Kvantum Institute, University of Oulu, Oulu, Finland

**Keywords:** DLST, Blood glucose, Type 2 diabetes, Glutamate metabolism, Keratinocytes, Sweat

## Abstract

To investigate the link between skin features and diabetes, we performed a multi-modal study combining a diabetic mouse model, a human pilot cohort, and keratinocyte response assays. Our goal was to identify molecular signatures in skin, cells, and sweat that correlate with glycemic control and could serve as early biomarkers of hyperglycemia. This integrative approach advances understanding of diabetes-associated dermatological changes and supports the development of targeted, non-invasive diagnostic tools for early detection.

Following glucose tolerance tests, proteomic and metabolomic profiling was performed. We identified dihydrolipoyl-succinyltransferase (DLST) as a promising biomarker whose protein and transcript levels consistently correlated with glycemic status across models. In HaCaT keratinocytes, DLST was found to drive metabolic reprogramming toward glutamine utilization as part of an antioxidant protective response. Chromatin immunoprecipitation assay revealed that Sp1 binds to the DLST promoter region, while functional studies, including Sp1 overexpression and knowdown, further confirmed that Sp1 regulates DLST at transcriptional level. Furthermore, DLST knockdown reproduced metabolic shifts in glutamine pathways, findings supported by proteomic analyses and stable isotopic flux analysis.

Reduced DLST expression in healthy skin corresponded with glutamine metabolism changes observed in keratinocytes upon oral glucose ingestion. While healthy skin and sweat displayed opposing metabolic trends, pre-type 2 diabetes samples showed aligned trends, suggesting sweat metabolomics could serve as an early systemic metabolic indicator.

These findings identify DLST as a glucose-responsive biomarker reflecting skin and sweat responses to systemic glucose, providing a foundation for the development of targeted, non-invasive diagnostic tools for early detection of diabetes.

## Highlights

• Identified DLST as a novel glucose-responsive biomarker in skin and its keratinocytes linked to systemic glycemic status.
• Demonstrated DLST-driven metabolic reprogramming in keratinocytes, shifting energy metabolism toward glutamine utilization.
• Showed opposing metabolic trends in healthy skin and sweat that converge in pre-type 2 diabetes.
• Demonstrated Sp1 binding to the DLST promoter and its role in regulating DLST transcription under high-glucose conditions.

## Graphical abstract

**Figure:**
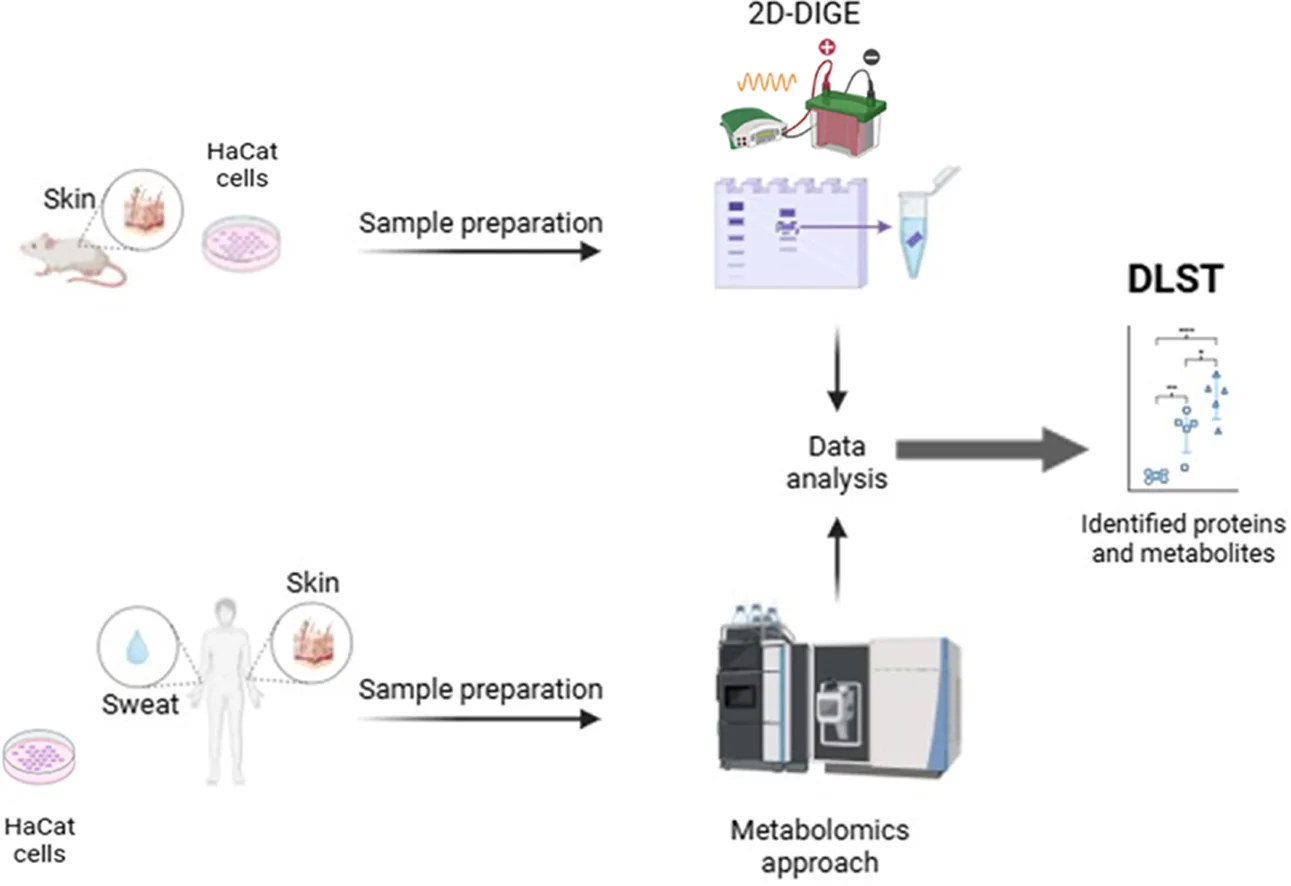

**Integrative Proteomic and Metabolomic Profiling of Skin, Sweat, and HaCaT Cells to Elucidate DLST-Associated Pathways**. This study combines proteomic and metabolomic approaches to investigate the role of DLST in skin physiology and metabolic regulation. Human skin biopsies, sweat samples, and HaCaT keratinocyte cultures were prepared for analysis using standardized protocols. Proteomic profiling was performed via two-dimensional difference gel electrophoresis (2D-DIGE) to identify differentially expressed proteins, while metabolomic analysis was conducted using advanced mass spectrometry. Data integration revealed key protein and metabolite signatures linked to DLST expression, offering insights into its molecular functions and potential implications in skin-related metabolic processes.

## 1 Introduction

Skin is increasingly recognized as a dynamic organ that reflects systemic metabolic changes, including those associated with diabetes [[1], [2], [3]]. Despite its accessibility and rich cellular diversity, skin remains an underexplored target for identifying novel biomarkers of glycemic dysregulation. Emerging evidence suggests that keratinocytes, sweat, and skin undergo characteristic changes in diabetes [[4], [5], [6], [7], [8], [9], [10], [11]], making skin a promising yet neglected window for early diagnosis and disease monitoring).

Accumulating evidence suggests that skin cells actively sense and respond to systemic glucose fluctuations. We previously demonstrated that keratinocytes exposed to hyperglycemia exhibit increased expression of Trisk95, calcium signaling dysregulation, and mitochondrial remodeling [3]. These findings support the concept that the skin is not merely a passive barrier, but a dynamic metabolic interface influenced by glucose homeostasis. Furthermore, sweat offers a minimally invasive matrix to study metabolic patterns in diabetic subjects. Our prior studies were among the first to provide evidence linking skin metabolic function with systemic glucose regulation, opening new avenues to better understand this association [12,13].

Glucose metabolism is tightly linked to mitochondrial function. A key regulatory node within the TCA cycle is the α-ketoglutarate dehydrogenase complex (α-KGD), which connects energy metabolism with redox balance and glutamate biosynthesis [14]. The α-KGD belongs to the 2-oxo acid dehydrogenase family and is composed of three catalytic subunits: E1 (oxoglutarate dehydrogenase, OGDH), E2 (dihydrolipoyl-succinyltransferase: DLST), and E3 (dihydrolipoamide dehydrogenase, DLD), each present in multiple copies within the complex. This multienzyme assembly catalyzes the oxidative decarboxylation of α-ketoglutarate to succinyl-CoA in the tricarboxylic acid (TCA) cycle, playing a central role in mitochondrial energy production and metabolism [15]. The DLST gene was first identified and its protein product cloned by Ali G et al.*,* who isolated the full-length cDNAs from a human fetal brain library and mapped the gene to chromosome 14q24.3, and thereby establishing DLST as the E2 component of α-KGD [16]. Subsequent structural studies showed that DLST form the catalytic and structural core of α-KGD, assembling into a 24-subunit oligomer with octahedral symmetry and containing flexible lipoyl domains that interact with adjacent core domains [[17], [18], [19], [20], [21], [22]]. Whitin the α-KGD, DLST catalyzes the transfer of the succinyl group from the reduced lipoyl moiety to coenzyme A, generating succinyl-CoA and playing a central role in mitochondrial ATP production and metabolic flux through the TCA cycle [23,24].

DLST is broadly expressed across tissues and has been implicated in both mitochondrial and extramitochondrial functions [15,[23], [24], [25]]. Notably, homozygous loss of DLST is embryonically lethal in mice, likely due to the inability of respiration-impaired mitochondria to maintain ATP homeostasis [26]. In the brain, reduced α-KGD activity has been associated with diminished glucose uptake and metabolism, a phenomenon thought to initiate a cascade of events leading to neurodegeneration [[27], [28], [29], [30]]. Consistent with this, a mouse model generated by crossing an amyloid-depositing transgenic line with DLST heterozygous (+/−) mice demonstrated that DLST deficiency accelerated the onset of spatial learning and memory deficits highlighting its essential biological role [31]. Beyond its catalytic activity in α-KGD, DLST has recently been shown to influence nuclear epigenetic regulation, such as histone succinylation, in response to metabolic changes [32]. Despite its broad functional significance, the role of DLST in skin tissue and its potential involvement in glucose-induced metabolic adaptation in keratinocytes remain unexplored.

Previous studies have demonstrated that high-glucose conditions can promote the utilization of glucose as a primary carbon source for anaplerotic process in various cell types, thereby influencing the metabolic flux through glutamine-dependent pathways [[33], [34], [35], [36]]. While direct evidence in keratinocytes is still lacking, recent transcriptomic analyses reveal that high glucose induces significant changes in metabolic gene expression changes in these cells [37]. This underscores the need for deeper investigation into how glucose might regulate DLST activity and glutamine metabolism, potentially contributing to metabolic adaptation in skin and its keratinocytes.

To explore this, our study employed a translational approach combining a diabetic mouse model, a human pilot study, and *in vitro* keratinocyte assays to explore DLST's role in skin and sweat-based metabolic pattern. We found that systemic glucose elevation alters DLST expressions, triggering a metabolic shift in keratinocytes toward glutamate synthesis, regulated by the transcription factor Sp1 and confirmed by DLST knockdown. Proteomic and metabolomic analyses revealed changes in glutamine and antioxidant pathways, with altered metabolic patterns in skin and sweat samples from healthy and prediabetic subjects.

We demonstrate that DLST expression is acutely and chronically altered by systemic glucose elevation, and that this triggers metabolic reprogramming in keratinocytes, from ATP-generating oxidative metabolism to glutamate synthesis. This shift, confirmed through DLST knockdown experiments, appears to serve as a protective response under high-glucose conditions. These observations were further validated by stable isotopic flux analysis, which revealed that DLST deficiency increased glutamate derived carbon towards glutamate metabolism, indicating a preferential reliance on glutamate metabolism compared with shCTRL cells. Proteomic and metabolomic analyses supported these findings, showing alterations in glutamine and antioxidant-related pathways. Importantly, changes in DLST expression and associated metabolic profiles in skin samples mirrored those observed in keratinocytes. Interestingly, while healthy skin and sweat samples exhibited opposing metabolic trends, samples from pre-type 2 diabetic subjects showed converging patterns, suggesting that sweat metabolomics may provide an early systemic indicator of metabolic dysregulation. Finally, chromatin immunoprecipitation assays revealed a direct binding of Sp1 to the DLST promoter under high-glucose conditions. Functional studies further demonstrated that Sp1 regulates DLST expression at the transcriptional level.

Together, our findings position DLST as a promising molecular biomarker and metabolic regulator for minimally invasive early metabolic indicator of type 2 diabetes through skin and sweat matrices.

## 2 Materials and methods

### 2.1 Mouse model

Type 1 diabetes (T1D) was induced in mice via a single intraperitoneal injection of streptozotocin (STZ) after a week of individual housing and a 4-hour fast, with controls receiving a citrate buffer injection, and diabetes confirmed at a blood glucose level of ≥15 mmoL/l, as previously described [3].

### 2.2 Glucose tolerance test assay

Glucose tolerance assay was performed as previously described in Ali N et al., 2020 [3].

### 2.3 Study design

The controlled study, approved by the Northern Ostrobothnia Hospital District Ethics Committee (EETMK:5/2022), recruited 31 participants (14 pre-T2D, 17 healthy) based on specific inclusion and exclusion criteria. The experimental protocol, detailed in supplementary materials (Supplementary Fig. 1E), involved three steps after a 12-hour fast: measuring fasting blood glucose, collecting skin biopsies, skin tape, and sweat samples, and administering a 75 g oral glucose solution. Skin epithelium samples were collected using a D-Squame disc, while sweat was collected via iontophoresis using the Macroduct® system. All procedures adhered to ethical standards and participant safety protocols.

## 3 Results

### 3.1 DLST as a novel skin biomarker reflecting blood glucose dynamics in mouse and human models

Building upon our previous hypothesis that the skin functions as a dynamic organ reflecting systemic metabolic changes and harboring potential biomarkers correlated with blood glucose fluctuations [3], we previously identified Trisk95 as a glucose-responsive protein that modulates calcium signaling and induces mitochondrial alterations. To further investigate the skin's molecular responsiveness to glucose dynamics, we performed two-dimensional difference gel electrophoresis (2D-DIGE) followed by mass spectrometry (MS) on skin biopsies collected from healthy mice injected with glucose or control. This analysis identified 20 protein spots differentially expressed 45 min post-glucose administration (Figure 1A; Supplementary Figure 1A; and Supplementary Table 1). Among these, only one protein (spot 13) was significantly upregulated in both glucose-treated, healthy and T1D mice when compared to their controls, suggesting its potential as a glucose-responsive biomarker (Figure 1B). Mass spectrometry identified this protein as dihydrolipoyl-succinyltransferase (DLST), a key mitochondrial component of the α-KGD (Supplementary Tables 1 and 2).

**Figure 1:**
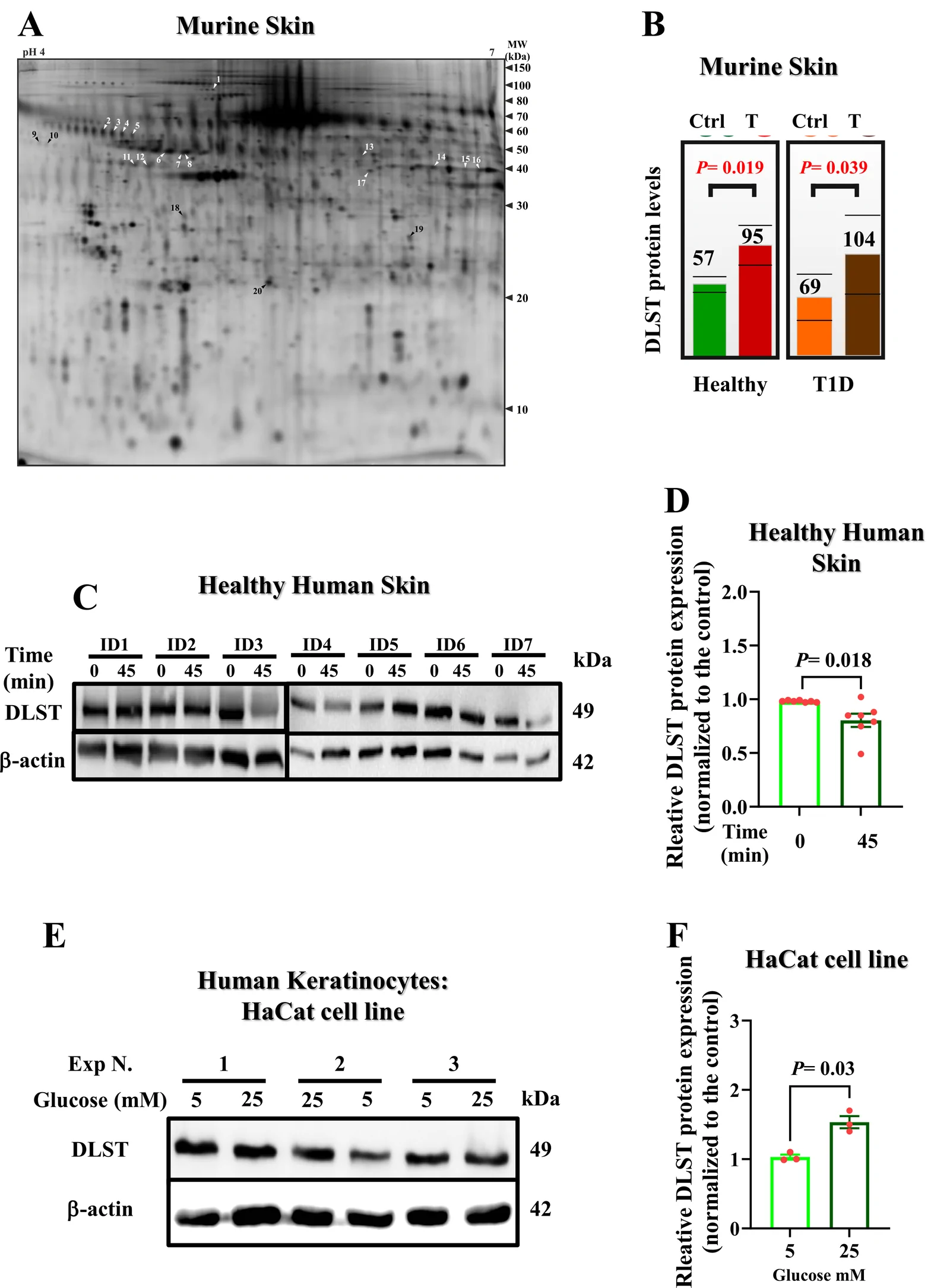
**Identification of DLST as a glucose-responsive protein in human and mouse skin. A.** Representative 2D-gel image of proteins (5 μg) from the skin of control (PBS)-injected type I diabetic mice. The proteins were labeled with Cy5 (saturation DIGE) and separated by IEF (pH4-7) and SDS-PAGE. Differentially expressed protein spots (1–20) are indicated. **B.** Expression profiles of DLST show the detected protein levels in healthy (H), and T1D (D) mice injected with PBS (control, Ctrl) or glucose (2 g/kg, T). Standard deviations are indicated. DLST identification with mass spectrometry was based on a MS score of 210, MSMS score of 43, as well as 9 and 4 covered peptides in the MS and MSMS analyses, respectively. **C.** Western blot analysis of DLST was performed on human skin samples from healthy individuals (n = 7), collected at fasting (0) and 45 min after glucose intake. β-Actin served as a loading control. **D.** Quantification of DLST expression in the human skin samples, normalized to fasting levels, showing decreased DLST in response to oral glucose intake in healthy skin (n = 7). **E.** Western blot analysis of DLST in HaCaT keratinocytes cultured in high glucose (25 mM) for at least three passages versus those maintained in physiological glucose (5 mM), using three independent experiments. **F.** Quantification of DLST levels in HaCaT cells confirms a significant downregulation in response to chronic high-glucose exposure. Data represent mean fold change ±SEM; significance was determined using a two-tailed Student's t-test, with *P* ≤ 0.05 considered statistically significant.

Western blot validation confirmed increased DLST levels in the skin of both glucose-injected healthy and T1D mice, relative to their respective controls (Supplementary Figure 1B–C, along with Supplementary Figures 6 and 7) (Table 2), supporting its relevance as a glucose-modulated biomarker. Because the skin comprises multiple cell types, we next assessed whether this regulation occurs specifically in keratinocytes. Primary keratinocytes isolated from healthy mice were cultured in normal glucose (5 mM) for 10 days, after which the medium was replaced with fresh medium containing either 5 mM or 25 mM glucose for 45 min, consistent with the *in vivo* experimental setting, as previously described [3]. RT-qPCR analysis revealed a significant elevation of *Dlst* mRNA expression in keratinocytes exposed to high glucose (Supplementary Figure 1D) (Table 3), consistent with the *in vivo* findings.

**Table 3:** Primers sequences used for quantitative RT-qPCR, qPCR, and ddPCR.

Target gene	Primer sequence (5′ to 3′)	Taq man assay ID ^TM^
***Sp1***	**F:** TGTGATCCCATGGCTACAGAG	
	**R:** GGCTGACTTCTTTGAGTGCCT	
**Tubulin**	**F:** GAGTGCATCTCCATCCACG	
	**R:** TAGAGCTCCCAGCAGGCATT	
***Dlst***		**TRCN0000344818**
***18S***		**Mm04277571_s1**
***GAPDH***		**Mm99999915_g1**

List of primer sequences used for quantitative RT-qPCR and ddPCR in this study. The primers corresponding to *Sp1* and *tubulin* were purchased from Sigma–Aldrich, and their sequences are provided below. Primers that target *Dlst*, *18S* and *GAPDH* were purchased from ThermoFisher as TaqMan assays, with their references number indicated. GAPDH = Glyceraldehyde-3-phosphate dehydrogenase.

Given the inherent limitations of 2D-DIGE, including restricted sample throughput, we employed a label-free proteomic approach for broader quantification. This revealed significantly lower DLST expression in T1D mouse skin compared to healthy controls (Supplementary Figure 1E), implicating insulin deficiency as a potential modulator of DLST levels in diabetic skin [3].

To translate these murine findings into human clinical context, we initially designed a study to recruit individuals with T1D, however, due to recruitment challenges, the study was refined into a pilot investigation to assess whether DLST expression in the skin correlates with systemic blood glucose levels. The revised study enrolled thirty-one healthy adult participants (22 females, 9 males; Table 1) underwent an oral glucose tolerance test (GTT) under resting conditions at room temperature. Participants were categorized based on fasting blood glucose levels into normoglycemic (3.9–5.5 mmol/L, n*=*17) and prediabetic (5.6–6.4 mmol/L, n*=*14) groups. Skin biopsies (abdominal region), epidermal layers (via skin tape), and sweat samples (via pilocarpine-iontophoresis) were collected at two time-points: fasting (baseline, B) and 45 min following glucose ingestion (post-intervention, A), as illustrated in the study workflow (Supplementary Figure 1F).

**Table 1:** Characteristics of participants in this study.

Group	Mean age ± SD (years) F/M	Mean BMI ± SD (kg/m^2^) F/M	Mean BG ± SD (mmol/L) F/M	Number F/M
Healthy controls	50.4 ± 8.4	25.1 ± 4.0	5.0 ± 0.4	15
	49.5 ± 21.9	26.0 ± 4.0	5.1 ± 0.3	2
Pre-T2D	59.7 ± 5.2	29.8 ± 4.7	5.9 ± 0.1	7
	49.4 ± 7.5	25.8 ± 1.6	6.2 ± 0.3	7

Data are presented as means ± SD. Participants were divided into two groups: Healthy controls (n = 17) and pre-T2D (n = 14). T2D = Type 2 Diabetes. BMI= Body Mass Index. BG. Blood glucose. F= Female. M = Male.

DLST expression in relation to blood glucose fluctuations was evaluated by western blot analysis of healthy skin collected from seven participants at fasting and 45 min after glucose intake. Western blot results revealed a consistent decrease in DLST protein levels 45 min after glucose ingestion (Figure 1C–D), in contrast to the increase observed in murine skin following glucose injection (Figure 1A–B and Supplementary Figure 5 and 6) (Table 2). This discrepancy likely reflects species-specific differences in glucose metabolism, particularly in the regulation of glucose transporters (Gluts) [38,39]. Consistent with our previous findings [3], Glut-1 expression was significantly elevated in the skin of T1D mice, accompanied with elevated glucose levels in both skin tissue and keratinocytes, despite markedly reduced insulin levels.

**Table 2:** Antibodies used for protein detection.

Immunogen	Company	Catalog nr.	Application	Dilutions
**DLST**	Novus biological	NBP1-85012	WB	1:500
**Sp1**	Santa cruz	Sc-17824	WB	1:500
**IgG**	Cell signaling	2729	Chip	1:500
**β-actin**	Sigma	A5441	WB	1:2000

List of antibodies used in this study. DLST = dihydrolipoyl-succinyltransferase. Sp1 = Specificity protein. IgG = Immunoglobulin G. b-actin = beta actin.

To further explore the relationship between prolonged blood glucose elevation and DLST transcriptional modulation, we assessed *DLST* mRNA levels in the epidermis (skin tape samples) at fasting from both normoglycemic and prediabetic individuals. qPCR analysis showed a significant upregulation of *DLST* mRNA in pre-T2D compared to healthy controls (Supplementary Figure 1G) (Table 3), suggesting that elevated blood glucose may transcriptionally induce *DLST* expression, potentially in keratinocytes; the predominant cell type in the epidermis (~92%) [40,41]. To test this hypothesis *in vitro*, we cultured human keratinocytes HaCat cells under normoglycemic (5 mM) and hyperglycemic (25 mM) conditions for three consecutive passages, consistent with established models of glucose exposure [37,[42], [43], [44], [45]]. Western blot analysis demonstrated a marked increase in DLST protein expression in cells maintained in high-glucose conditions (Figure 1E,F along with Supplementary Figure 7) (Table 2). Given that the α-KGD consists of three subunits [15], we next examined the protein expression levels of the remaining two subunits, E1 and E3, using western blot analysis. No changes in the expression of either subunit were observed (Supplementary Figure 8, Supplementary Figure 9, and Supplementary Figure 10). These findings align with the *DLST* upregulation observed at the mRNA level in pre-T2D epidermis and support the hypothesis that keratinocytes contribute to the glucose sensitive regulation of DLST.

Collectively, our results demonstrate that skin exhibits a dynamic, glucose-responsive molecular landscape, with DLST emerging as a promising biomarker candidate for monitoring systemic glucose fluctuations in both mice and humans.

### 3.2 High glucose conditions enhance Sp1 binding to the DLST promoter and regulate DLST transcription in HaCat cells

Building on our clinical observation that *DLST* mRNA levels are elevated in the epidermis of pre-T2 individuals compared to healthy controls, we sought to investigate the molecular mechanism underlaying this regulation using the *in vitro* model. Specifically, we aimed to determine whether high glucose influences *DLST* expression at the transcriptional level in keratinocytes and identify the key transcription factors involved in this response. To conclude, we performed RT-qPCR analysis on HaCat cells cultured under normal–or high-glucose conditions. Consistent with the clinical data, *DLST* mRNA levels were significantly upregulated in cells exposed to high glucose (Figure 2A) (Table 3), indicating transcriptional regulation of *DLST* in response to hyperglycemia.

**Figure 2:**
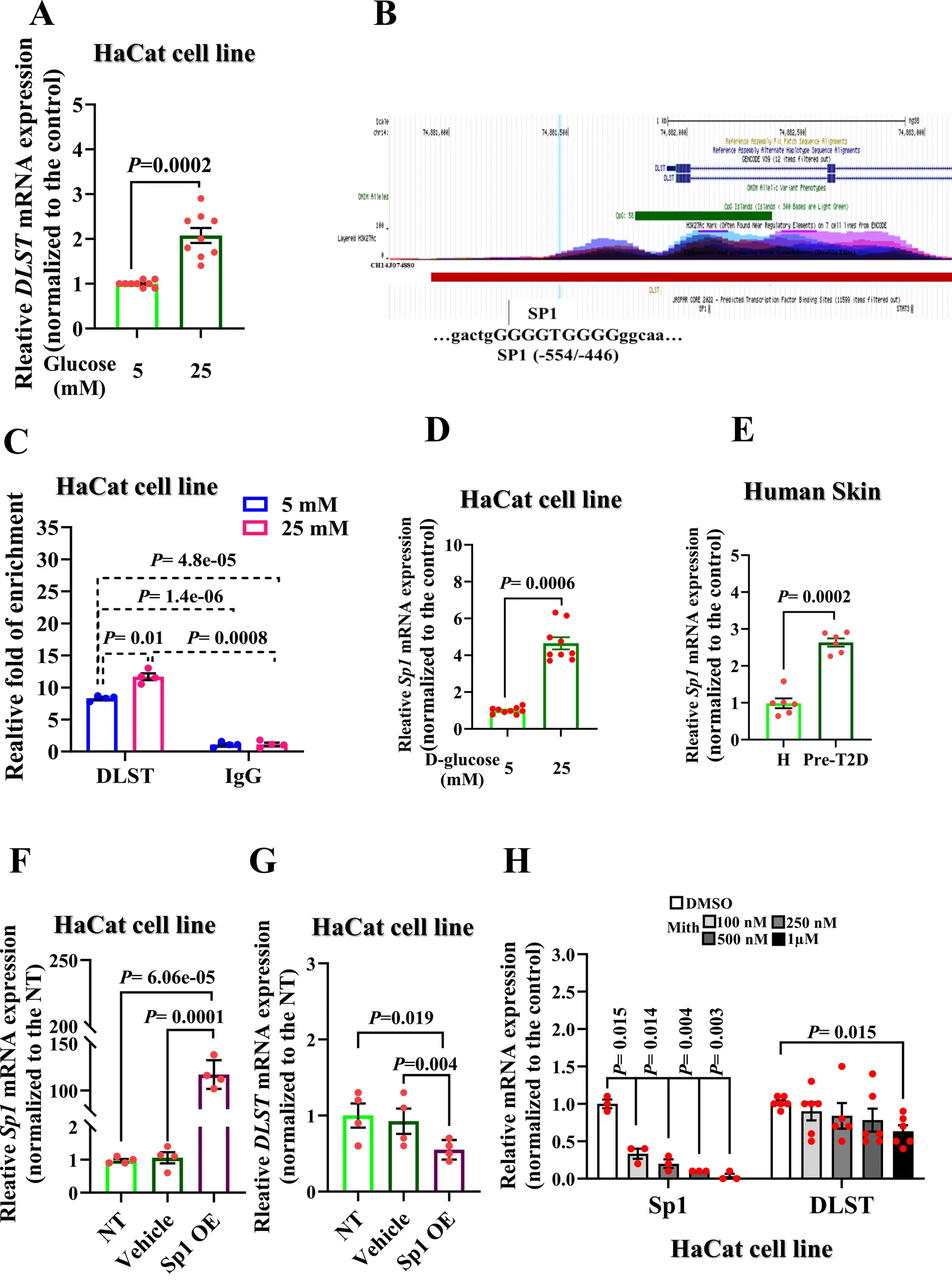
**High glucose exposure regulates DLST transcription through Sp1 promoter binding. A.***DLST* mRNA expression was measured by RT-qPCR in HaCaT cells cultured under normal (5 mM) or high (25 mM) glucose conditions. Results are shown as mean ± SEM from three independent experiments, normalized to control values. **B.** A schematic Representation of the *DLST* gene and its promoter from the UCSG genome browser highlights a putative Sp1-binding site (GGGGTGGGG) located upstream of the translational start codon (ATG). **C.** ChIP assays were performed using anti-Sp1 antibody on keratinocytes maintained with normal (5 mM) or high glucose (25 mM). The enrichment of *DLST* promoter fragments at two specific sites (positions 77 and 99) was quantified by qPCR. Data are expressed as mean ± SEM from three biological replicates performed in triplicate. **D.***Sp1* mRNA expression levels were analyzed in HaCat cells maintained under normal or high glucose conditions using RT-qPCR. **E.***Sp1* mRNA expression levels were measured in the epidermis of healthy and pre-T2D individuals at fasting using RT-qPCR. Each condition included samples from three individuals, with all measurements performed in triplicate. **F.***Sp1* mRNA expression levels were quantified by qPCR following Sp1 overexpression (Sp1 OE) in HaCat cell line. **G.***DLST* mRNA expression levels were quantified by qPCR following Sp1 overexpression (Sp1 OE) in HaCat cell line. In panels **F** and **G**, each condition included four biological replicates, with all measurements performed in triplicate. **H.***Sp1* and *DLST* mRNA expression levels were quantified by qPCR following pharmacological inhibition of Sp1 activity with mithramycin in HaCaT cells. Each condition included three to six biological replicates, and all samples were analyzed in technical triplicate. NT, non-transduced cells; Vehicle, cells transduced with the empty pcDNA3 vector; Sp1 OE, cells transduced with the full-length human Sp1 cDNA expression plasmid (pcDNA3-Flag-Sp1). DMSO, dimethyl sulfoxide, used as the vehicle control; Mith, mithramycin, used as a specific inhibitor of Sp1 activity; nM, nanomolar; μM, micromolar. Statistical analysis was performed using Two-tailed Student's t-test. *P* ≤ 0.05 was considered statistically significant.

To explore the upstream regulatory mechanisms, we performed an *in-silico* analysis using the UCSC genome databases (http://genome.ucsc.edu/) to identify potential transcription factor binding sites within the *DLST* promoter. This analysis revealed a consensus Sp1-binding motif as a potential transcription factor for *DLST* promoter activity. We examined the presence of Sp1-binding motif (5ʹ-GGGGTGGGG-3ʹ) within the *DLST* promoter region (Figure 2B), suggesting that Specificity Protein 1 (Sp1) may be involved in mediating glucose-induced *DLST* transcription. We next tested whether Sp1 directly binds to the *DLST* promoter using chromatin immunoprecipitation (ChIP) assays in HaCaT cells cultured under 5 mM or 25 mM glucose conditions. RT-qPCR analysis of the immunoprecipitated chromatin demonstrated a significant enrichment of Sp1 binding to the GC-rich promoter region of *DLST* in cells exposed to high glucose compared to those maintained in normal glucose (Figure 2C) (Table 4). These findings support the direct binding of Sp1 to the DLST promoter under high glucose conditions; however, further investigations are needed to confirm its functional role.

**Table 4:** Primers sequences used for quantitative ChIP RT-qPCR.

Target gene	Primer sequence (5′ to 3′)
***Sp1***	**F:** 5′-TGTTGTTGTGATCCCATGGCT-3′
	**R:** 5′-GAGTGCCTCCTTCTCCTTCAC-3′
***Dlst***	**F:** 5′-TTAGGGTTGCCTGCTCCAGA-3′
	**R:** 5′-GTGTTCGGAGCGAACCACT-3′
**Negative control**	**F:** 5′-AAGCCAGGATTTGTGCTGGG-3′
	**R:** 5′-TTCAAGCTGCCCGAGAAAGCG-3′

List of primer sequences used for quantitative ChIP RT-qPCR in this study.

To determine whether glucose influences Sp1 expression itself, we analyzed *Sp1* mRNA levels under prolonged high-glucose exposure. Results showed a significant upregulation of *Sp1* in keratinocytes maintained in high-glucose conditions compared to normal-glucose controls (Figure 2D) (Table 3). This finding is in agreement with previous reports demonstrating elevated Sp1 levels in the skin of diabetic patients relative to healthy individuals [46]. Building on these findings, we next examined whether sustained elevations in blood glucose levels influence *Sp1* expression in human skin. To this end, we performed qPCR analysis on epidermal RNA isolated from skin tape samples collected at fasting from both normoglycemic and pre-T2D individuals. *Sp1* mRNA levels were significantly elevated in the prediabetic group compared to healthy controls (Figure 2E) (Table 3), suggesting that sustained hyperglycemia may transcriptionally upregulate *Sp1* expression in the epidermis. This pattern closely mirrors the expression profile of *DLST*, reinforcing the proposed regulatory axis linking systemic glucose levels, *Sp1* activation, and *DLST* induction in human skin.

To investigate whether Sp1 regulates DLST transcription under high-glucose conditions, we modulated Sp1 expression and activity using two complementary approaches: SP1 overexpression (SP1 OE) through transfection with a full-length human SP1 cDNA expression plasmid, and pharmacological inhibition of SP1 transcriptional activity using the specific SP1 inhibitor mithramycin [[47], [48], [49]]. Following these interventions, Sp1 and DLST expression levels were evaluated at the mRNA level by qPCR at 48 h after Sp1 overexpression and 24 h after Mith treatment. Introduction of the full-length Sp1 cDNA using the pcDNA3 expression plasmid (Sp1 OE), as previously described [50], resulted in a significant increase in *Sp1* mRNA expression compared with vehicle-treated or non-transduced control (NT) cells (Figure 2F). Interestingly, Sp1 overexpression was accompanied by a significant reduction in *DLST* mRNA expression (Figure 2G) (Table 3), raising the possibility that Sp1 may participate in a feedback regulatory mechanism or interacts with additional transcriptional regulators, such as HIF-1α, to influence DLST expression under metabolic stress conditions, as discussed in the Discussion. To further examine the role of Sp1 activity in regulating DLST expression, HaCaT cells were treated with increasing concentrations of Mith (100 nM, 250 nM, 500 nM, and 1 μM), with DMSO (final concentration 0.1 %) used as the vehicle control. Mithramycin treatment resulted in a significant, gradual, dose-dependent reduction in *Sp1* mRNA expression (Figure 3H) (Table 3). Similarly, DLST expression followed a comparable trend, with significant modulation observed particularly at the 1 μM Mith concentration.

**Figure 3:**
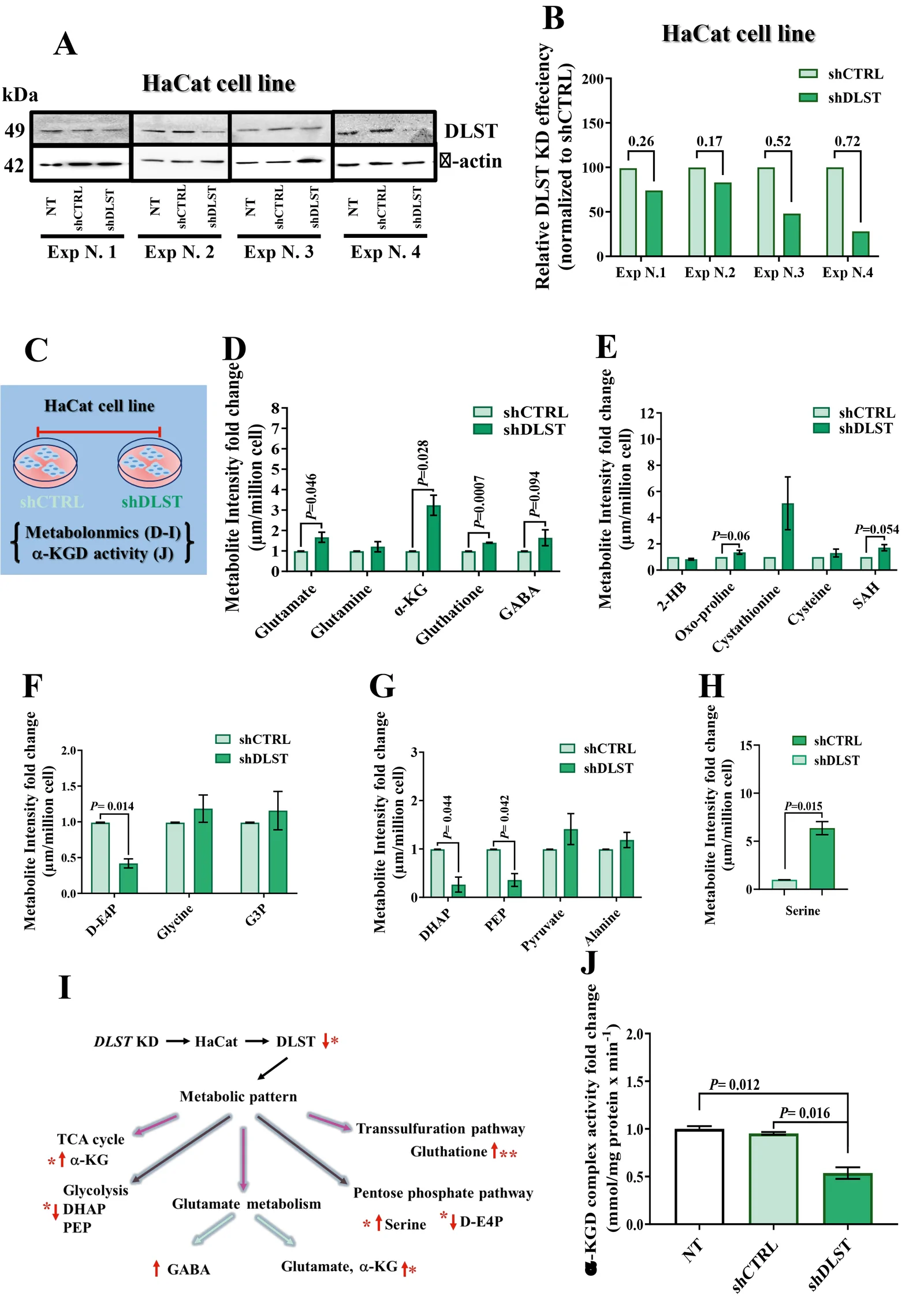
**Moderate DLST Knockdown reveals its role in metabolic reprogramming under high-glucose conditions in keratinocytes. A.** Western blot analysis confirmed DLST knockdown in HaCaT cells using lentiviral shRNA (shDLST), compared to non-transduced (NT) and vehicle-transduced control cells (shCTRL), across four independent experiments. **B.** Quantification showed DLST protein levels reduced by 26%, 17%, 52%, and 72% in knockdown cells. β-Actin served as the loading control. **C.** Schematic overview of the experimental design: shCTRL and shDLST cells were maintained under high-glucose conditions (25 mM) and subsequently subjected to metabolomic profiling and enzymatic activity analyses. **D-H.** Targeted metabolomics revealed altered patterns in key pathways in shDLST versus shCTRL cells under high glucose, using data combined from three independent moderate DLST KD cell lines. The affected pathways include glutamate metabolism (**D**), transsulfuration (**E**), pentose phosphate pathway (**F**), glycolysis (**G**), and serine biosynthesis (**H**). Data are peak area fold change ±SEM of three independent experiments after normalization to the control. **I.** A graphical summary illustrates the metabolic pathways influenced by DLST silencing in 25 mM conditions. **J.** α−KGD activity was significantly reduced in DLST knockdown cells under high glucose compared with shCTRL cells. Data are presented as mean ± SEM from three independent experiments performed in triplicate and normalized to total protein concentration. Statistical comparisons were conducted using a two-tailed Student's t-test, with *P* ≤ 0.05 considered statistically significant. 2-HB: 2-hydroxybutyrate; SAH: S-adenosylmethionine. D-E4P: d-erythrose-4-phospahte. G3P: Glycerol 3-phosphate. DHAP: Dihydroxyacetone phosphate. PEP: Phosphoenolpyruvate.

Together, these findings position Sp1 as an important transcriptional regulator contributing to DLST modulation under high-glucose conditions. Across both experimental and clinical settings.

### 3.3 Altered DLST expression drives metabolic shifts in keratinocytes

Building on both basic and clinical evidence highlighting DLST as a glucose-responsive protein, and considering its central role in mitochondrial metabolism as a component of the α-KGD [15,24], we sought to investigate whether alterations in DLST expression influence the metabolic profile of keratinocytes under high glucose conditions. Clinically, DLST protein levels were found to decrease following acute glucose intake in both normoglycemic and prediabetic individuals, raising the question of whether this downregulation leads to functional metabolic consequences.

To this end, we generated a DLST knockdown (DLST KD) HaCaT cell line (referred to as shDLST) using lentiviral shRNA transduction across four independent passages. Non-transduced cells (NT) were used to confirm that the control lentivirus did not alter DLST expression in shCTRL cell line. Given the unaltered DLST expression in shCTRL compared to NT, as shown in the western blot images (Figure 3A), we proceeded with band quantification using only shCTRL and shDLST cells. Western blot analysis confirmed variable knockdown efficiencies of 26%, 17%, 52%, and 72 % (Figure 3B) (Table 2). These cell lines were subsequently subjected to a targeted metabolomics analysis under high-glucose conditions to assess changes in key metabolic intermediates and evaluate potential shifts in metabolic pathways associated with DLST suppression, as illustrated in the representative schematic (Figure 3C). Preliminary analysis of metabolomic profiles across the four experiments revealed that shDLST cells with moderate DLST downregulation (26%, 17%, and 52%) exhibited consistent metabolic patterns (Figure 3C–I and Figure 4A–I). In contrast, the experiment with higher DLST downregulation (Exp.4; 72%) displayed a distinct and opposing metabolic profile (Figure 4A–E). Given our aim to model the biologically relevant range of DLST downregulation observed *in vivo* (13–49%), statistical analyses were performed using only the first three experiments (Exp. 1–3), which fell within this range. Exp. 4 represented by a single biological replicate, was not included in the statistical analysis but is presented as a representative example of the metabolic pattern associated with more pronounced DLST depletion and to provide comparison with the moderate DLST knockdown conditions (Figure 4).

**Figure 4:**
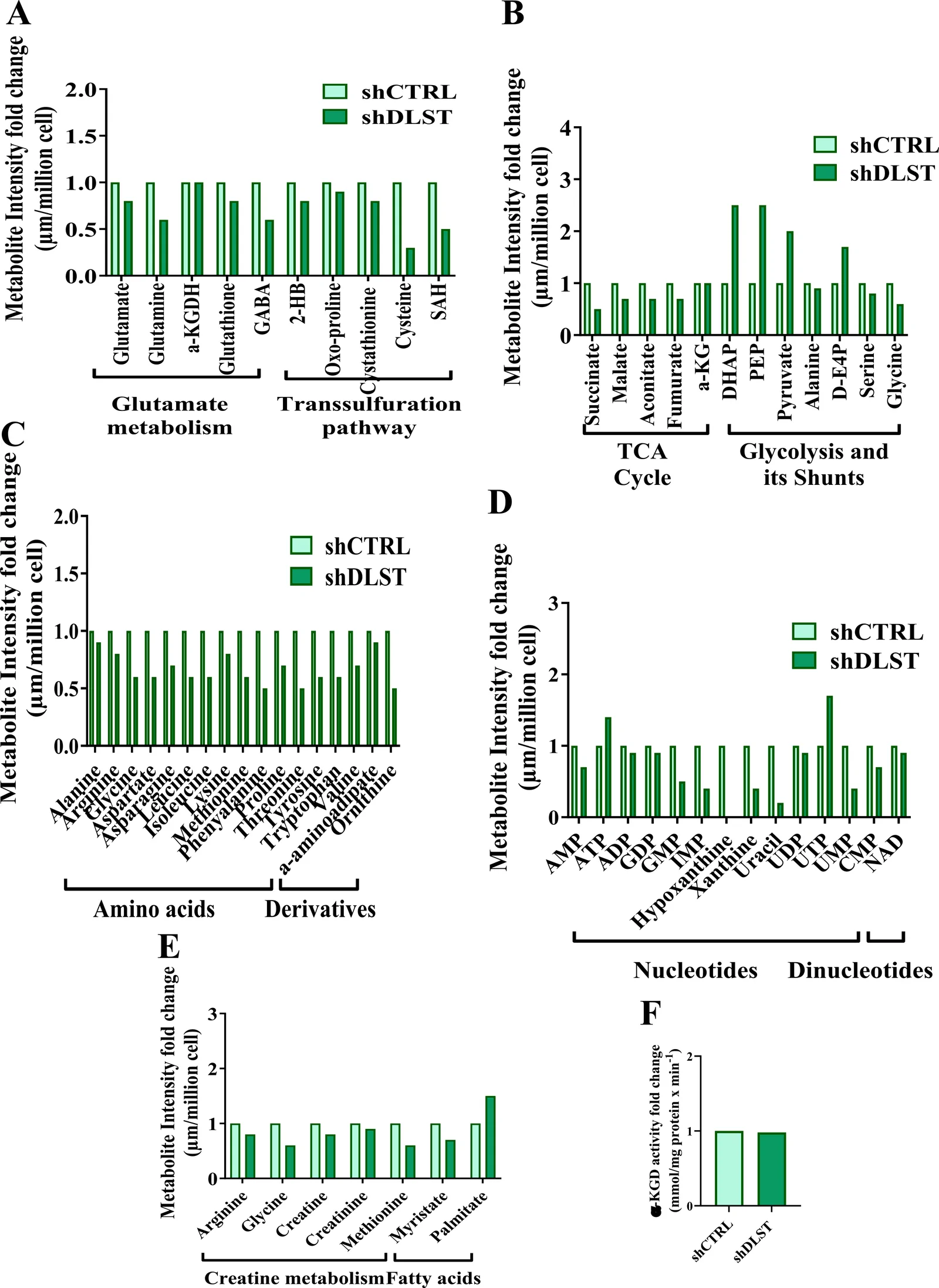
**High DLST Knockdown induces a distinct metabolic profile under high-glucose conditions. A-E.** Targeted metabolomics analysis demonstrated alterations in multiple metabolic pathways in shDLST cells (72 % knockdown) relative to shCTRL cells under high-glucose conditions. These include glutamate metabolism and the transsulfuration pathway (**A**), the TCA, glycolysis and associated shunts (**B**), amino acid metabolism and related derivatives (**C**), nucleotide and dinucleotide metabolism (**D**), and creatine and fatty acids (**E**). **F.** α−KGD activity remained unchanged in shDLST cells (72 % knockdown) under high glucose compared with shCTRL cells. α-KG: alpha ketoglutarate. 2-HB: 2-hydroxybutyrate. GABA: Gamma-aminobutyric acid. SAH: S-adenosylmethionine. DHAP: Dihydroxyacetone phosphate. PEP: Phosphoenolpyruvate. D-E4P: d-erythrose-4-phospahte. G3P: Glycerol 3-phosphate. AMP: Adenosine monophosphate. ATP: Adenosine triphosphate. ADP: Adenosine diphosphate. GDP: Guanosine diphosphate. GMP: Guanosine diphosphate. IMP: Inosine monophosphate. UDP: Uridine diphosphate. UTP: Uridine triphosphate. UMP: Uridine monophosphate. CMP: Cytidine monophosphate. NAD: Nicotinamide adenine dinucleotide.

Compared to shCTRL cells, shDLST cells exhibited a notable increase in metabolites associated with glutamate metabolism, specifically α-KG, glutamate, and glutathione, reflecting the functional connection of DLST to the α-KGD (Figure 3D). While a trend towards increased GABA levels was observed (Figure 3D and Supplementary Figure 2A), the elevated glutathione suggests potential activation of transsulfuration pathway (Figure 3E). In contrast, glycolytic intermediates such as dihydroxyacetone phosphate (DHAP) and phosphoenolpyruvate (PEP) were significantly reduced (Figure 3F,G). Furthermore, moderate DLST knockdown resulted in marked upregulation of serine (Figure 3H), alongside elevations in tyrosine and valine (Supplementary Figure 2H). Interestingly, xanthine was significantly decreased (Supplementary Figure 2B). While modest elevations were also observed in certain nucleotides, redox cofactors, creatine, fatty acids, and amino acid derivatives metabolites, these did not reach statistical significance (Supplementary; Figure 2C–I). In contrast, the fourth experiment (72% knockdown) exhibited a divergent metabolic profile characterized by reduced glutamate metabolism, as well as decreased activity across the transsulfuration pathway, TCA cycle, amino acid, nucleotide and nucleotide-derivative metabolism, and creatine metabolism (Figure 4A–E). In parallel, glycolytic intermediates were increased, accompanied by a trend toward elevated ATP and UTP levels (Figure 4B,D, and E), suggesting a shift toward glycolysis as the primary energy source rather than glutamate metabolism observed in the three conditions. Notably, no changes were observed in α-KG levels (Figure 4A) or α-KGD activity (Figure 4F), indicating a distinct metabolic adaptation under higher DLST suppression. These findings may suggest a potential non-canonical or compensatory role of DLST under conditions of stronger knockdown, which is further discussed in the Discussion section. However, as this observation is based on a single experimental replicate, further experiments are required to confirm this finding.

To further explore the protein-level changes associated with moderate DLST deficiency in the three cell lines used for metabolomic analysis, we performed 2D-DIGE electrophoresis to identify differentially expressed proteins in DLST-deficient HaCat cells culture under high-glucose conditions. The analysis revealed a substantial reduction in both DLST (spot 160) and the E1 component of the α-KGD (E1; spot 14) (Figure 5A–C). These findings confirm the knockdown at the proteomic level and support the observed *in vitro* reduction in α-KGD activity (Figure 3J). A marked decrease was also detected in glutamine-hydrolyzing isoform (GMP synthase; spots 88 and 89), an enzyme that converts glutamine to glutamate (Figure 5A, D-E). This downregulation may partially explain the observed trend of increased glutamine and suggests compensatory upregulation of glutamate through alternative mechanisms, such as transamination involving α-KG. This mechanism may reflect anaplerotic replenishment of the TCA cycle. The concurrent rise in amino acid levels further supports the notion that glutamine metabolism becomes a predominant energy source under these conditions. In addition, we observed a trend toward increased expression of glutathione S-transferases (GSTs; spots 361, 390, and 359) and serine/arginine rich splicing factor 1 (SRSF1; spot 294) in DLST knockdown cells compared with shCTRL controls (Figure 5F–J). These protein profiles are consistent with the elevated metabolites glutathione and serine, since glutathione functions as a key antioxidant in redox homeostasis [51,52], and SRSF1 is involved in RNA splicing and may be regulated indirectly by serine availability [53,54].

**Figure 5:**
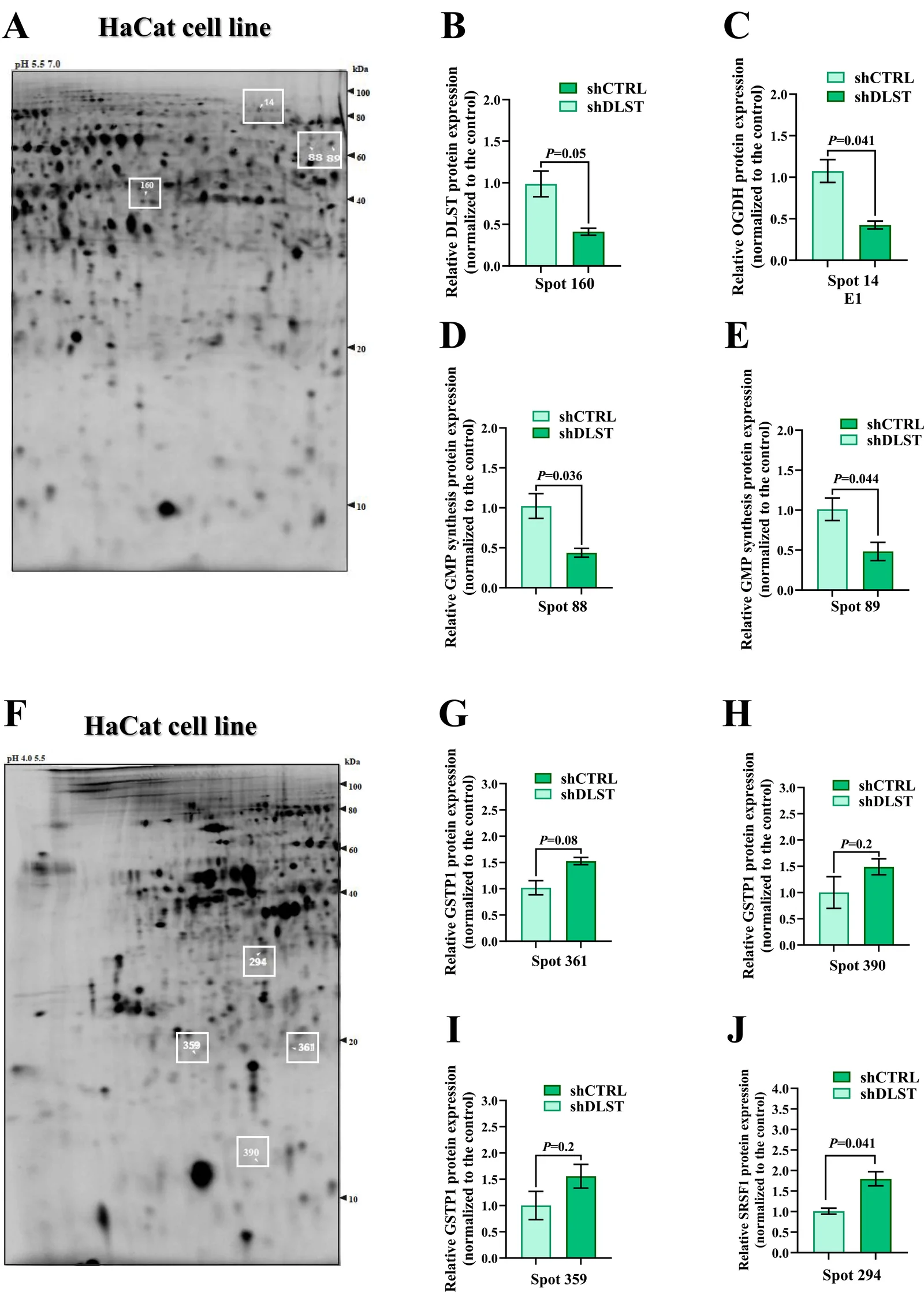
**DLST Knockdown alters protein expression profiles linked to metabolic remodeling in keratinocytes. A.** Representative 2D-DIGE image (right panel) of the HaCat cells (shCTRL, 5 mM glucose), highlighting statistically significant protein spots: 361, 390, 359 and 89. Proteins (5 μg) were labelled with Cy5 (saturation DIGE) and separated by IEF (pH4-7) and SDS-PAGE. Notably spot 160 corresponds to DLST, spot 14 to E1 subunit, and spots 88 and 89 to GMP synthase. **B-E**. Quantifive analysis of selected protein expression levels shDLST cells compared to shCTRL: DLST (**B**), E1 (**C**), and GMP synthase (**D**–**E**). **F.** Representative 2D-DIGE image (left panel) of HaCat cells (shCTRL, 5 mM glucose) highlighting protein spots 361, 390, 359, and 294, which show a trend of increased expression following DLST knockdown. Spots 361, 390, and 359 correspond to glutathione S-transferases (GSTs), while spot 294 represents SRSF1 (serine/arginine rich splicing factor 1). Data are presented as mean ± SEM from three independent experiments and normalized to total protein concentration. Statistical comparisons were conducted using a two-tailed Student's t-test, with *P* ≤ 0.05 considered statistically significant.

Collectively, these findings suggest the activation of a protective metabolic adaptation aimed at maintaining redox homeostasis during prolonged high-glucose exposure, at least in part through a DLST-dependent manner.

### 3.4 DLST KD modulates carbon flux in keratinocytes under different glucose conditions

To further confirm that reduced DLST directs the α-ketoglutarate towards glutamate pathways under high-glucose conditions, we performed stable isotopic flux analysis in shCTRL and shDLST cultured in 25 Mm glucose. Additionally, to examine the effect of high glucose concentration alone on the glutamate metabolism, shCTRL cells maintained in 5 mM or 25 mM glucose were also analyzed. Cells were then incubated with ^13^C5 l-glutamine for 2 h, and the incorporation of ^13^C5 into various central carbon intermediates was then subsequently assessed, as illustrated in the schematic protocol (Figure 6A).

**Figure 6:**
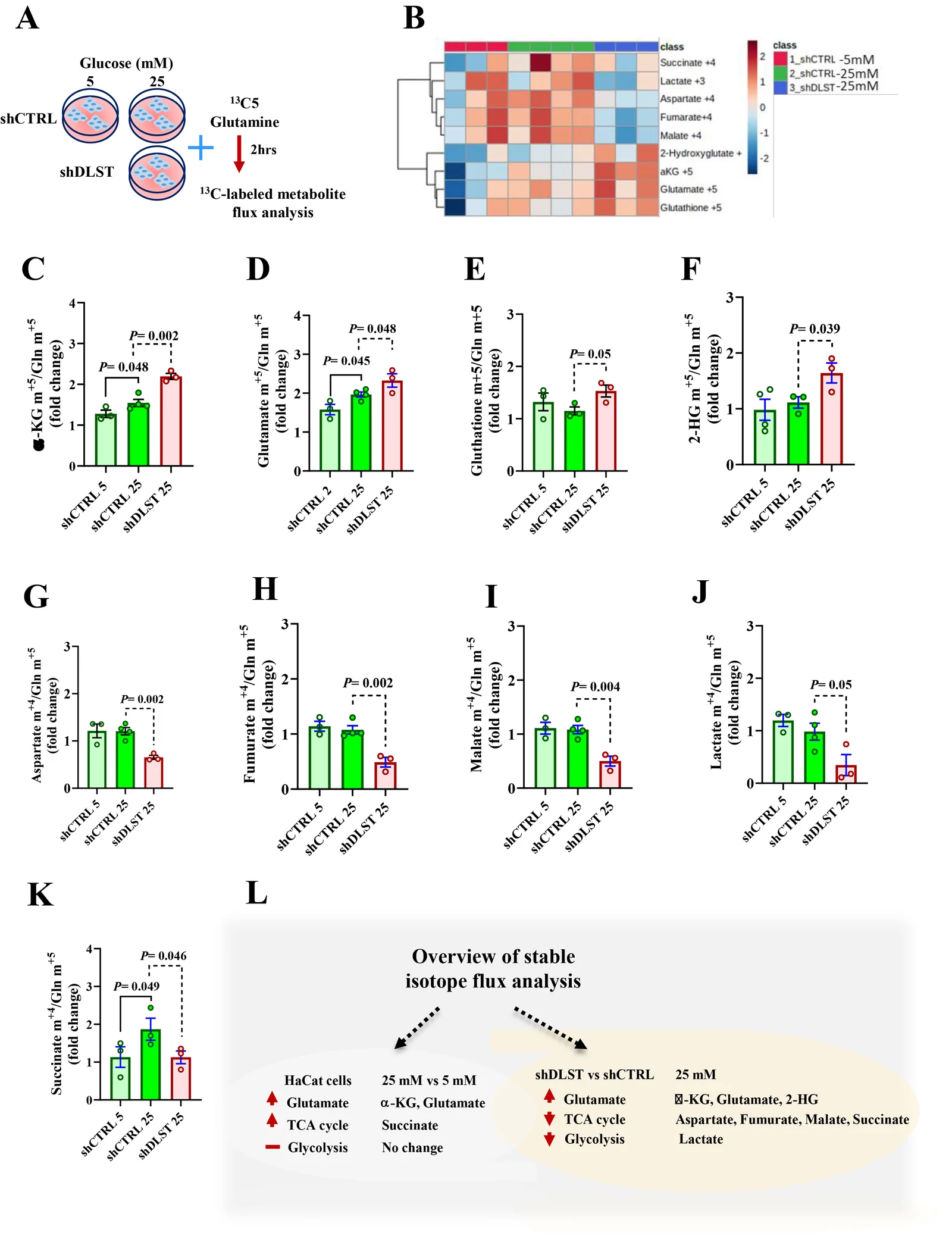
**DLST knockdown and high glucose conditions alter carbon flux in keratinocytes. Α.** Schematic representation of the experimental strategy. ShCTRL and shDLST cells were incubated in presence of media containing ^13^C5 l-glutamine for 2 h, followed by downstream isotope flux metabolic analysis. **B.** Heatmap of the metabolic profiles of shCTRL cells under 5 mM or 25 mM glucose, alongside shDLST cells under 25 mM glucose conditions. **C–K.** Ratios of labelled metabolites to glutamine: α-KG m^+5^/Gln m^+5^ (**C**), glutamate m^+5^/Gln m^+5^ (**D**), glutathione m^+5^/Gln m^+5^ (**E**), 2-HG m^+5^/Gln m^+5^ (**F**), aspartate m^+4^/Gln m^+5^ (**G**), Fumarate m^+4^/Gln m^+4^ (**H**), Malate m^+4^/Gln m^+5^ (**I**), Lactate m^+4^/Gln m^+5^ (**J**), and succinate m +4/Gln m+5 (**K**)**. L.** Overview of labelled carbon trafficking between different pathways. Data are ratio fold changes ±SEM of three or four biological replicates. Statistical analysis was performed using a two-tailed Student's t-test, with *P* ≤ 0.05 considered statistically significant. 2-HG: 2-hydroxyglutarate.

Our findings are illustrated in a heatmap, which displays the measured intermediates and highlights significant differences between shDLST and shCTRL cells under high-glucose conditions, as well as between shCTRL cells maintained in normal and high glucose conditions (Figure 6B). Specifically, in cells cultured in 25 mM glucose, DLST deficiency led to a significant increase in the α-KG m^+5^/Gln m^+5^, glutamate m^+5^/Gln m^+5^, and 2-HG m^+5^/Gln m^+5^ ratios compared with shCTRL cells (Figure 6C–D and F). A trend towards increased glutathione m^+5^/Gln m^+5^ ratio was also observed in shDLST cells (Figure 5E). In contrast, the ratios of TCA cycle intermediates, including aspartate m^+4^/Gln m^+5^, fumarate m^+4^/Gln m^+5^, malate m^+4^/Gln m^+5^, and succinate m^+4^/Gln m^+5^, as well as the glycolysis intermediate lactate m^+4^/Gln m^+5^ were significantly decreased (Figure 6G–K). Collectively, these results indicate that shDLST cells may utilize glutamate metabolism as an energy source more than control cells.

Importantly, when comparing the metabolic flux in shCTRL cells maintained in high versus normal glucose, a pattern similar to that observed in DLST deficiency cells was noted. Specifically, α-KG m^+5^/Gln m^+5^ and glutamate m^+5^/Gln m^+5^ ratios were substantially increased relative to 5 mM (Figure 6C–D). This was accompanied by an opposite pattern of succinate m^+4^/Gln m^+5^ ratios to that observed in shDLST, which showed a significant elevation relative to 5 mM glucose (Figure 6K). However, no changes were observed in glutathione, 2-HG, aspartate, fumarate, malate, or lactate ratios (Figure 6E–J), indicating carbon shunting from the TCA cycle to glutamate metabolism in response to high glucose.

Collectively, our results support a key role for DLST in orchestrating carbon flow between the different energy pathways, emphasizing its function as an adaptive mechanism to maintain keratinocyte metabolic homeostasis under varying glucose concentrations (Figure 6L).

### 3.5 Dynamic blood glucose levels modulate skin and sweat metabolic profiles

Based on our hypothesis that the skin is a dynamic organ to reflect internal metabolic states and may serve as a source of biomarkers associated with blood glucose fluctuations, we sought to clinically translate our experimental findings. Specifically, we aimed to investigate whether alterations in blood glucose levels influence the metabolic profiles of skin and sweat, and whether these profiles exhibit comparable patterns. This effort was further motivated by our *in vitro* observations, which identified DLST as a key regulator of glucose-induced metabolic shifts in human keratinocytes. To address this, we conducted a targeted metabolomics analysis on skin and sweat samples collected from both healthy and pre-T2D individuals. A panel of 41 metabolites spanning multiple metabolic pathways was examined to assess potential differences between the two participant groups.

In healthy skin participants (n = 6), we assessed changes in 41 targeted metabolites in the skin following glucose intake. When comparing metabolite levels at 45 min post-oral glucose ingestion to those at fasting, we observed a significant increase in α-KG, GABA, and 2-hydroxybutyrate (2-HB) (Figure 7A–C), accompanied by elevated levels of glutamate and glutamine (Supplementary Fig. 3A and B). Additionally, creatinine levels were substantially upregulated, alongside increased carnitine (Figure 7D, and Supplementary Figure. 3C, respectively). Arginine and lactate also showed significant increases (Figure 7E,F). Notably, high-glucose intake led to elevated levels of the fatty acid palmitate, myristate, and linoleate in the skin (Figure 7G–I).

**Figure 7:**
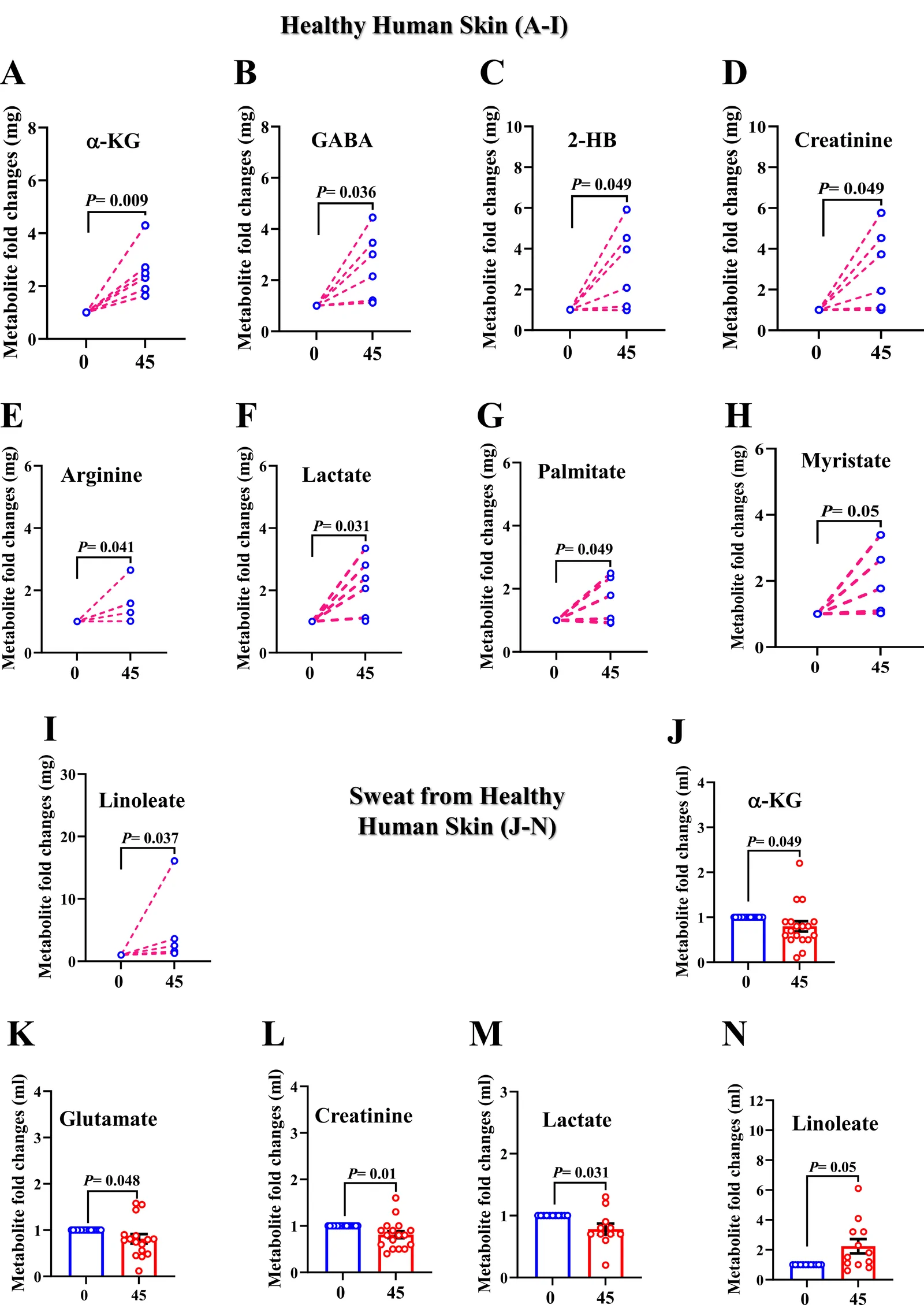
**Blood glucose levels influence metabolic profiles in human skin and sweat. A-I.** Targeted metabolomics analysis revealed that fluctuations in blood levels significantly alter the metabolic composition of human skin and sweat in healthy individuals. Panels **A-I** show changes in skin metabolite (n = 8) 45 min after oral glucose ingestion, under conditions of elevated blood glucose levels. Significant changes were observed in metabolites including α-KG (**A**), GABA (**B**), 2-HB (**C**), creatinine (**D**), arginine (**E**), lactate (**F**), palmitate (**G**), myristate (**H**), and linoleate (**I**). Panels **J-N** present sweat metabolite profiles from healthy subjects (n = 11–18), also 45 min post-glucose intake, highlighting alterations in α-KG (**J**), glutamate (**K**), creatinine (**L**), lactate (**M**), and linoleate (**N**). Data are expressed as fold change in peak area ±SEM of eight independent participants for skin samples and 11–18 independent participants for sweat samples, normalized to baseline (control) values. Statistical analysis was performed using a two-tailed Student's t-test, with *P* ≤ 0.05 considered statistically significant.

In contrast, the metabolic profile of sweat samples (n = 11–18) revealed an inverse pattern in response to elevated blood glucose intake showed significant decreases in α-KG, glutamate, creatinine, and lactate compared to fasting levels (Figure 7J-M). Interestingly, linoleate was the only metabolite significantly increased in sweat under high-glucose conditions (Figure 7N), suggesting its potential as a non-invasive biomarker for blood glucose monitoring.

In pre-T2D participants (n = 3), analysis of the 41 targeted metabolites revealed that twenty metabolites were decreased in skin samples collected 45 min after oral glucose ingestion compared to fasting levels. Among these, ten metabolites exhibited statistically significant downregulation under high-glucose conditions (Figure 8A–J), while the remaining ten showed a similar downward trend without reaching statistical significance (Supplementary Figure. 4A–J). In sweat samples from the same pre-T2D participants, the metabolic profile shared limited overlap with the changes observed in the skin. Notably, both α-KG and glutamine levels were reduced in sweat at 45 min post-glucose intake, though only α-KG reached statistical significance (Figure 8K,L).

**Figure 8:**
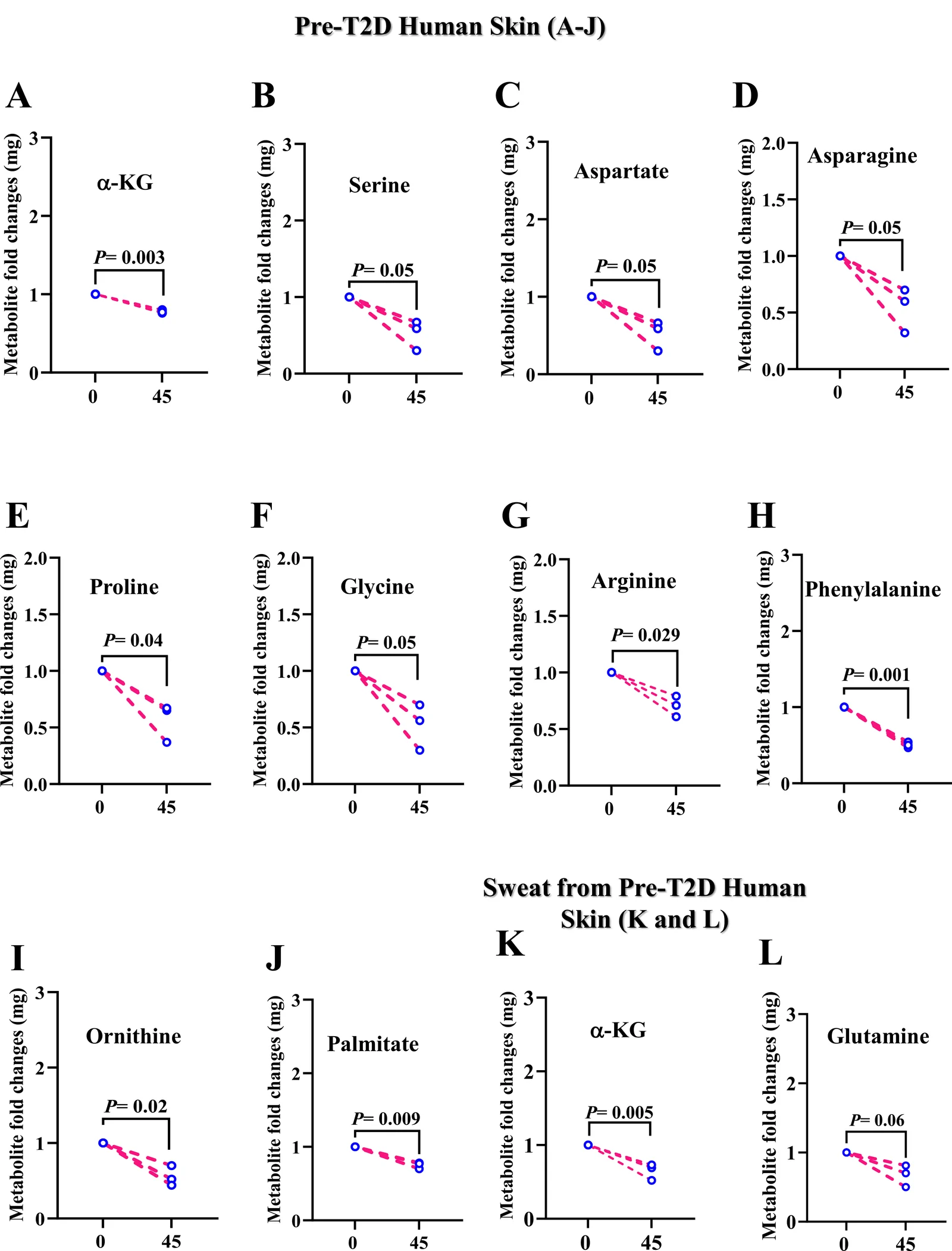
**Blood glucose levels influence metabolic profiles in human skin and sweat in pre-T2D individuals. A–J.** Targeted metabolomics analysis revealed that elevated blood glucose levels significantly alter the metabolic composition of human skin in individuals with prediabetes (n = 3), measured 45 min after glucose intake. Notable changes were observed in metabolites including α-KG (**A**), serine (**B**), aspartate (**C**), asparagine (**D**), proline (**E**), glycine (**F**), arginine (**G**), phenylalanine (**H**), ornithine (**I**), and palmitate (**J**). **K** and **L.** Sweat metabolite profiles from pre-T2D subjects (n = 3), also collected 45 min post-glucose intake, showed alterations in α-KG (**K**) and glutamine (**L**). Data are expressed as fold change in peak area ±SEM from three independent participants for both skin and sweat samples, normalized to baseline (control) levels. Statistical comparisons were performed using a two-tailed Student's *t*-test, with *P* ≤ 0.05 considered statistically significant.

To further investigate whether elevated blood glucose levels characteristic of the pre-T2D state influence skin metabolism differently from normoglycemic conditions, we performed a comparative analysis of the 40 targeted metabolites in skin samples collected at fasting from both pre-T2D and healthy participants. The results revealed a general trend toward increased metabolite levels in the pre-T2D group, with ornithine showing a statistically significant increase, except aconitate, E4P, and linoleate that showed a trend of decrease (Supplementary Table 3).

In healthy individuals, skin and sweat exhibited opposite metabolic responses following glucose intake, suggesting that sweat may serve as a buffering role for the skin. Moreover, the metabolic profile observed in healthy skin aligns with *in vitro* findings in keratinocytes, particularly with respect to glutamine metabolism, potentially linked to DLST downregulation. Conversely, in pre-T2D subjects, the metabolic profiles of skin and sweat were more similar, implying a loss of this buffering capacity. This dysfunction may indicate early impairment of sweat gland function, a feature commonly reported in type 2 diabetes and could potentially serve as an early diagnostic indicator for disease onset.

Our findings indicate that blood glucose fluctuations are linked to specific metabolic changes in skin and sweat, with glutamate pathway metabolites as potential biomarkers for non-invasive glucose monitoring. However, these results need validation in larger, diverse cohorts due to the small sample size.

## 4 Discussion

This report is the first to show proof-of-concept evidence highlighting DLST as a key orchestrater of metabolic responses in both experimental and clinical settings under systematic glucose fluctuations, as summarized in Figure 9. We highlight a potential metabolic shift with significant implications for clinical translation, particularly for the early detection of type 2 diabetes. Importantly, this metabolic reprogramming reveals the dynamic adaptation of human keratinocytes to excess glucose, positioning the skin as a critical organ for deeper investigation in biomarker discovery. Understanding the biological mechanisms behind such biomarkers, including previously identified Trisk 95 ^3^, and currently DLST, is crucial.

**Figure 9:**
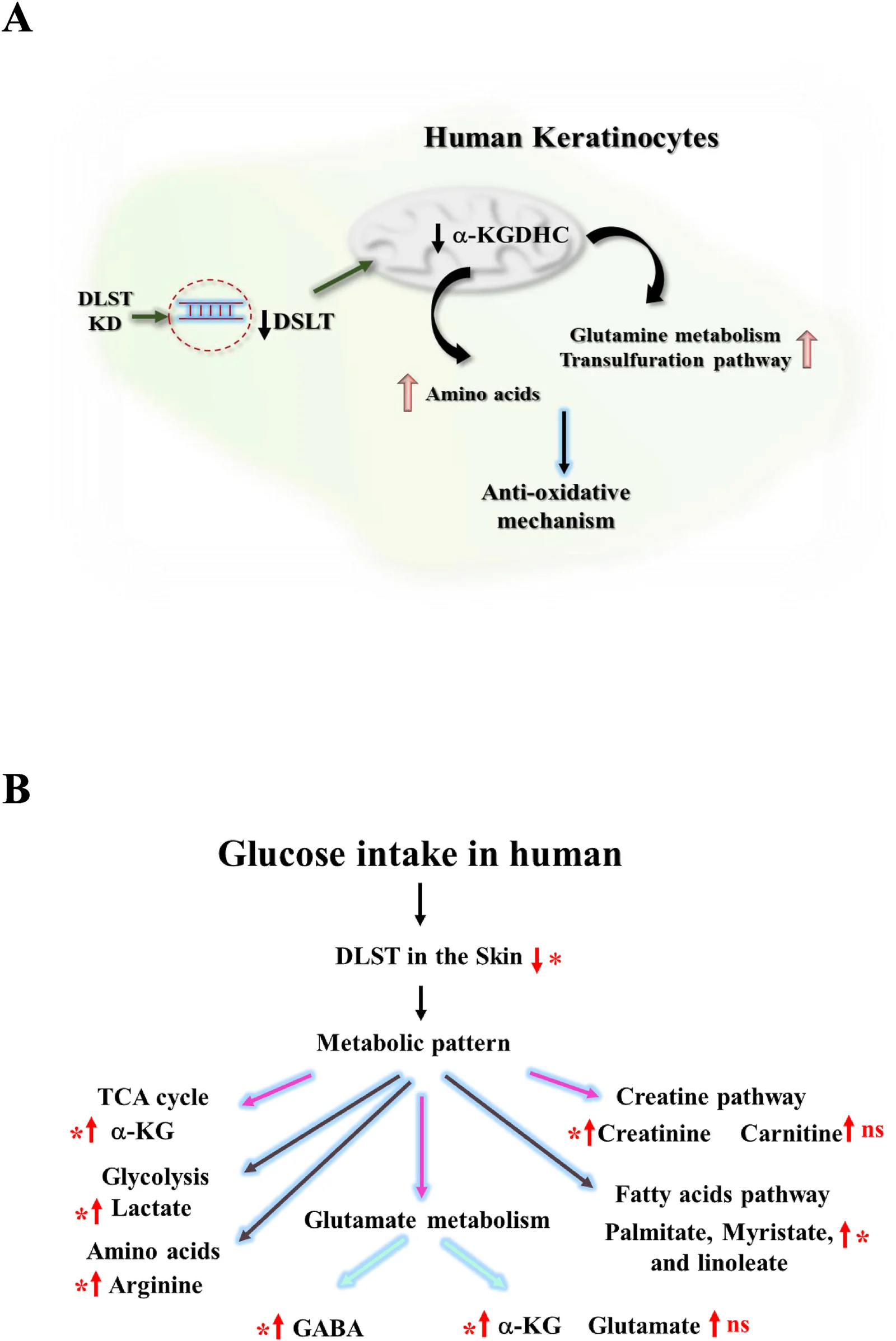
**Proposed role of DLST in modulating glucose-responsive metabolic reprogramming in keratinocytes and humans skin**. **A.***In vitro* model: We propose a model in which DLST serves as a central regulator of metabolic reprogramming in human keratinocytes under hyperglycemic conditions. DLST knockdown in keratinocytes cultured in high-glucose conditions results in reduced activity of the α-KGD, redirecting metabolism toward enhanced glutamine utilization. This shift promotes elevated levels of glutamine-derived intermediates, including α-KG, glutamate, glutathione, and metabolites of the transsulfuration pathway. Additionally, it is associated with reduced xanthine production and increased levels of various amino acids and nucleotides metabolites. These alterations suggest that glutamine metabolism becomes a major source of carbon and energy under metabolic stress. **B.***In vivo* clinical model: In human subjects, glucose intake results in a downregulation of DLST expression at both the transcriptomic and proteomic levels. This suppression is accompanied by widespread metabolic alterations, including increased activity in glutamine metabolism, the TCA cycle, creatinine metabolism, fatty acid pathways, glycolysis, and amino acid metabolism. Notably, several of these metabolic shifts parallel those observed in DLST-deficient keratinocytes, suggesting that DLST may play a pivotal role in orchestrating systemic metabolic responses to elevated blood glucose levels.

Although DLST has not been previously studied in skin tissues, its downregulation has been reported in neurodegenerative diseases [[55], [56], [57]]. Notably, the complete loss of DLST results in embryonic lethality in mice, likely due to impaired mitochondrial respiration and the failure to maintain ATP homeostasis [26]. In the brain, reduced α-KGD activity has been associated with diminished glucose uptake and metabolism, a phenomenon thought to initiate a cascade of events leading to neurodegeneration [[27], [28], [29], [30]]. Supporting this, DLST^+/−^ mouse model has been generated, showing normal development and brain morphology but exhibiting significant reduction in α-KGD activity in the brain [58]. Our data derived from three human keratinocytes cell lines with moderate DLST knockdown align with these findings, showing that shDLST cells exhibit decreased α-KGD activity compared to shCTRL cells under high-glucose conditions. This is further supported by our results demonstrating that DLST knockdown is accompanied by reduced E1 subunit expression, as observed via 2D-DIGE electrophoresis and in agreement with previous reports [59,60].

However, this relationship may not always be linear, as prior experimental and clinical studies have reported discrepancies between protein expression levels and enzyme activity, including α-KGD deficiency without reduced protein levels [[61], [62], [63], [64]]. Consistent with this, in a fourth shDLST cell line, a higher degree of DLST knockdown did not result in a corresponding decrease in α-KGD activity, nor in change in α-KG metabolite levels. One possible explanation is that DLST plays a major, yet uncharacterized, non-canonical in human keratinocytes under high-glucose conditions, potentially exerting a more prominent function than the other two subunits (E1 and E3). In support of this, high-glucose conditions alone decreased DLST levels without altering the expression of E1 and E2 in HaCat cells, further underscoring its central role in maintaining α-KGD integrity and activity. Its limited availability within the α-KGD complex may reflect a balance between its canonical enzymatic function and its involvement in adaptive responses to altered glutamate metabolism or increased oxidative stress. This may involve a feedback mechanism that modulates α-KGD complex activity and its integration within the TCA cycle. Supporting this notion, our ongoing work demonstrates that DLST may interact with cytoplasmic proteins involved in oxidative stress and exhibit nuclear localization in keratinocytes maintained with high-glucose conditions, whereas this localization is not observed in normal glucose conditions (Mäkelä M et al., unpublished), consistent with previous findings by Wang et al. [32].

Our findings highlight the clinical relevance of α-KG metabolism as a potential biomarker. Both our stable isotopic flux analysis and clinical observations both indicated an increase in α-KG levels associated with reduced DLST protein levels in two scenarios: prolonged high glucose and in shDLST cells under high-glucose conditions. This finding is particularly relevant in light of previous clinical observations showing that reduced α-KG levels in plasma and liver tissues were correlated with higher HbA1c and increased insulin resistance in patients [[65], [66], [67]], underscoring the potential role of α-KG as an early indicator of diabetic complications. Moreover, our data indicate that decreased DLST levels, reduced α-KGD activity, and accumulation of α-KG coincide with a metabolic shift toward glutamate metabolism under hyperglycemic conditions, as further supported by stable isotopic flux analysis. Consequently, glutamate-derived carbon becomes a major anaplerotic replenishing the TCA cycle, suggesting that keratinocytes adopt an adaptive anaplerotic strategy to maintain metabolic homeostasis [14,68,69]. This observation aligns with the reductive carboxylation pathway of glutamine metabolism [70], commonly observed under hypoxia, a condition present in our keratinocyte model (Motei et al., unpublished). Reductive carboxylation has been described as the conversion of glutamine into α-KG to support oxaloacetate and acetyl-CoA production [70,71]. In shDLST cells cultured under high-glucose conditions, we observed increased NAD(H) pool, possibly due to anaplerosis-driven serine biosynthesis as a metabolic feedback loop, as previously reported [69,72,73]. These cells also exhibited significantly elevated levels of serine, along with increased alanine and aspartate, compared to shCTRL cells. Based on these findings, we speculate that DLST-driven glutamate may serve as a source of generating of non-essential amino acids (NEAA), including serine, alanine, and aspartate via transaminases, thereby supporting cellular biosynthesis, as previously reported [74].

Our clinical data revealed an additional metabolic feature associated with increased glutamate metabolism and decreased DLST expression in the skin of healthy subjects. Specifically, we observed significant increases in fatty acids, including palmitate, myristate, and linoleate following oral glucose intake. These findings are consistent with prior reports linking glutamine metabolism to lipid and *de novo* nucleotide synthesis [71,75]. Similarly, a comparable trend towards increased levels of these fatty acids was observed in pre-T2D participants compared with healthy controls, further supporting a role of glutamine in lipid synthesis. Although metabolomics of prediabetic skin is sparse, blood metabolomics in prediabetic patients show increased palmitate and myristate, a nonsignificant decrease in linoleate, elevated amino acids like tyrosine and phenylalanine, and decreased glutamine [76]. These literature observations partially align with the metabolic patterns observed in our pre-T2D skin participants, who exhibited increased fatty acid metabolites and decreased linoleate. In contrast, we observed increased glutamate metabolism in our pre-T2D skin compared with blood data, likely reflecting tissue-specific metabolic adaptations. Furthermore, sweat metabolomics in pre-T2D participants showed patterns parallel to those in the skin when comparing 45 min post–glucose intake with fasting levels, particularly for linoleate that showed a reduced levels in both matrices. In contrast, healthy subjects displayed opposite trends in most metabolites between skin and sweat under the same conditions. Together, these coordinated metabolic changes in skin and sweat provide clinical and novel insights that may aid in the early detection and prevention of prediabetes. Notably, one participant reverted from prediabetic to normoglycemic status following dietary intervention during the study, underscoring the potential life-saving impact of such measures (Ali N et al., unpublished).

High-glucose conditions in keratinocytes elevate reactive oxygen species (ROS) production [[77], [78], [79]], which keratinocytes counteract by increasing glutathione synthesis [80]. Given glutathione's role in neutralizing xanthine, a known oxidative stress marker [[81], [82], [83]], and our observed decrease in xanthine in shDLST cells, our data suggest a protective mechanism mediated by elevated glutathione levels. Stable isotopic flux analysis further confirmed these findings, showing an increased glutathione m^+5^/Gln m^+5^ ratio in shDLST under high-glucose conditions. Similarly, previous reports demonstrated that keratinocytes can cope with highly induced antioxidative stress by upregulating glutathione levels [84,85]. Supporting this, our proteomics analysis revealed a trend toward increased GSTP1 expression and decreased GMP synthase patterns in shDLST compared with shCTRL cells. Both enzymes were reported to facilitate glutathione recycling and synthesis, thereby maintaining cellular redox balance [52,86,87]. Clinically, the relevance of glutathione was underscored by observations that lower glutathione levels in T2D patients were associated with microvascular complications, including nephropathy and retinopathy [[88], [89], [90]]. Based on our data and clinical observations, we suggest that DLST may represent a potential therapeutic target in diabetes, particularly for preventing or mitigating skin-related complications associated with oxidative stress through a glutathione mediated mechanism. Another supportive mechanism involves GABA levels, which were also observed in our cellular and clinical models. GABA is expressed in keratinocytes localized in the superficial layers of human epidermis and is associated with keratinocytes differentiation [91]. In addition, GABA plays a major role in cell protection against oxidative stress produced by UV radiation [92,93].

In our experimental model, Chip-qPCR and functional experiments demonstrated that Sp1 binds to the DLST promoter and contributes to the regulation of DLST transcription. Interestingly, Sp1 overexpression resulted in reduced *DLST* mRNA levels, whereas pharmacological inhibition of Sp1 activity produced a similar reduction in both *Sp1* and *DLST* mRNA expression, suggesting a context-dependent regulatory relationship between Sp1 activity and DLST regulation. This observation raises the possibility that Sp1 may participate in a feedback mechanism or interact with additional transcriptional regulators to control DLST expression. Given the reported interaction between Sp1 and HIF-1α [[94], [95], [96], [97], [98]], and previous studies demonstrating that HIF-1α can modulate Sp1-dependent transcriptional programs through interaction with promoter-bound Sp1 complexes [99,100], HIF-1α may represent an additional regulatory component influencing DLST expression under metabolic stress conditions. Supporting this hypothesis, our supplementary analysis (Supplementary Fig. 11) showed that HIF-1α expression was increased following Sp1 overexpression and reduced after pharmacological inhibition of Sp1 activity, supporting a potential Sp1/HIF-1α regulatory interplay in keratinocytes. This interaction may be particularly relevant under chronic hyperglycemic conditions, where high-glucose exposure has been associated with HIF-1α activation and hypoxia-like responses in keratinocytes (Mäkelä et al., unpublished). Furthermore, the increased Sp1 expression observed in diabetic skin compared with healthy controls in both human and rat models [46] supports the potential involvement of this axis in metabolic adaptations during diabetic skin pathology. Beyond Sp1 and HIF-1α, other transcriptional regulators may contribute to DLST regulation. For example, a recent study reported reduced DLST protein levels following MYC depletion in MYC-driven T-cell acute lymphoblastic leukemia, suggesting a potential MYC-dependent regulatory relationship with DLST [101]. In the same study, DLST was shown to contribute to TCA cycle reprogramming, including modulation of α-KG levels, which is consistent with the metabolic patterns observed in our current study. Additionally, keratinocytes express Sp3, a transcription factor that shares the same GC-rich promoter binding sites with Sp1, suggesting another potential layer of transcriptional control. Collectively, these observations highlight the complexity of DLST regulation and support future investigation of crosstalk among Sp1, HIF-1α, and MYC, and Sp3 in mediating DLST responses to glucose fluctuations.

Collectively, our study unveils a previously unrecognized metabolic signature in skin and sweat, positioning DLST as a molecular “early warning clock” for prediabetes. To our knowledge, this is the first evidence identifying DLST as a functional glucose sensor in the skin, linking its regulation to distinct metabolite patterns. These findings pave the way for the development of novel diagnostic approaches capable of detecting subtle metabolic shifts long before the onset of overt diabetes, as well as therapeutic strategies to address skin-related manifestations associated with diabetes.

## CRediT authorship contribution statement

**Nsrein Ali:** Writing – review & editing, Writing – original draft, Validation, Supervision, Methodology, Investigation, Formal analysis, Data curation, Conceptualization. **Syeda Tayyiba Rahat:** Writing – review & editing, Investigation, Data curation. **Diana Motei:** Writing – review & editing, Validation, Investigation, Formal analysis. **Mira Mäkelä:** Writing – review & editing, Investigation, Formal analysis. **Subodh Sharma:** Investigation, Visualization, Writing – review & editing. **Mikko Tulppo:** Writing – review & editing, Methodology. **Juhani Juntilla:** Writing – review & editing, Methodology. **J. Kalervo Hiltunen:** Writing – review & editing, Methodology, Investigation. **Anni I. Nieminen:** Writing – review & editing, Methodology, Formal analysis. **Steffen Ohlmeier:** Writing – review & editing, Methodology, Formal analysis, Data curation. **Seppo Vainio:** Writing – review & editing, Supervision, Resources, Funding acquisition, Conceptualization.

## Ethics declaration

Written informed consent to take part in the study and to publish the article has been obtained from all participants or their legal representatives. The privacy rights of participants have been observed.

This study included organ or tissue donors. This study includes human biological material and consent was obtained by donors, or their next of kin or legal representatives, for use in this study and for publication of the article. The samples used in this research were not sourced from executed prisoners or prisoners of conscience.

This study was performed in compliance with relevant laws, regulatory frameworks and guidelines where the research took place. This study was approved by the Northern Ostrobothnia Hospital District Ethics Committee. (Approval No. EETMK:5/2022).

This study was conducted in accordance with the following guidelines for animal welfare and/or reporting: European Convention (ETS 123), EU Directive 86/609/EEC. This study was approved by the National Animal Experiment Board (ELLA).

## Informed consent

Applicable, informed consent was obtained for all human sample collections.

## Resource availability statement

No applicable resources were generated or analyzed during the current study.

## Approval of the research protocol

Applicable, this study involved both human and animal subjects.

## Approval date of registry and registration No

Applicable, this study received ethics approval for the human pilot study under reference number EETMK:5/2022.

## Animal study

Applicable, this study involved animal models. All the animal procedures were performed in accordance with the European Convention (ETS 123), EU Directive 86/609/EEC, and the Finnish national legislation, as approved by the local ethical committee.

## Declaration of competing interest

The authors declare that they have no known competing financial interests or personal relationships that could have appeared to influence the work reported in this paper.

## Data Availability

No data was used for the research described in the article.
