# Supplementary material for: DLST-driven metabolic alterations link skin to blood glucose: A translational study from mouse to human
Source: Mol Metab. 2026 Sep 28;114:102439. doi: 10.1016/j.molmet.2026.102439 (PMC13682495; doi:10.1016/j.molmet.2026.102439)
Supplement: Supplementary file 1 [file mmc1.docx]

**DLST-driven metabolic alterations link skin to blood glucose: A translational study from mouse to human**

**Nsrein Ali ^(1, 2, 3, 4) #^, Syeda Tayyiba Rahat ^(1)^, Diana Motei ^(1)^, Mira Mäkelä ^(1)^, Subodh Sharma ^(4)^, Mikko Tulppo ^(5, 6)^, Juhani Juntilla ^(5, 6)^, J. Kalervo Hiltunen ^(1)^, Anni I Nieminen ^(7)^, Steffen Ohlmeier ^(8)^, Seppo Vainio ^(1, 2, 3, 9)^**

^(1)^ Faculty of Biochemistry and Molecular Medicine, ^(2)^ Infotech Oulu, ^(3)^ Flagship GeneCellNano, ^(4)^ Faculty of Medicine, University of Oulu, Oulu, 90220, Finland. ^(5)^ Medical Research Center Oulu, Cardiology, Faculty of Medicine, University of Oulu. ^(6)^ Resaerch Unit of Biomedicine and Internal Medicine, Faculty of Medicine, University of Oulu. ^(7)^ Stem Cell and Metabolism Research Program, Faculty of Medicine, University of Helsinki, Finland. ^(8)^ Proteomics and Protein Analysis Core Facility, Biocenter Oulu, University of Oulu, Oulu, Finland. ^(9)^ Kvantum Institute, University of Oulu, Oulu, Finland.

\# To whom correspondence should be addressed. E-mail: [nsrein.ali@oulu.fi](mailto:nsrein.ali@oulu.fi)

**Address for Correspondence:**

Nsrein Ali. PhD.

Faculty of Biochemistry and Molecular Medicine

Faculty of Medicine

Laboratory of Developmental Biology

Aapistie 5A, FIN-90220,

University of Oulu,

Oulu, Finland.

Telephone: +358 (0) 50 39 94 212

ORCID. 0000-0001-9173-3717

**Supplementary materials**

**Mouse model**

**The type I diabetic mouse model**

T1D was induced after one single intraperitoneal (IP) injection of streptozotocin (STZ), as previously described ^1^. One week before the experiment, all mice were housed in individual cages. On the day of the experiment, they fasted for 4h and were then divided into two groups: controls and type I diabetic mice. The control mice were injected IP with Na citrate buffer (pH 4.5), while the type I diabetic mice were injected with STZ (150 mg/kg) diluted in Na citrate buffer to a total volume of 100 µl. The mice were considered as diabetic when their blood glucose level was ≥ 15 mmol/l.

**Glucose tolerance test assay**

Glucose tolerance assay was performed as previously described in Ali N *et al.,* 2020 ^1^. For adaptation purposes, the mice were housed in individual cages for one week. Prior to the experiment the healthy and T1D mice fasted overnight (12h) and each group was then divided into two parts, yielding four groups in all: healthy and T1D groups that were injected intraperitoneally (IP) with PBS, and healthy and type I diabetic groups that were injected IP with D-glucose (2g/kg). After 45 min, all the mice were humanely euthanized, and skin samples were collected for the various experiments (proteomics, western blot, and qRT-PCR).

**Two-dimensional difference gel electrophoresis (2D-DIGE) – Murine Skin and HaCat cell line**

For the proteomic study, purified skin tissues extracts (n=4 healthy and n=6 diabetic per group; control and treated) and HaCat cell line samples (n=3 per group) were purified and concentrated with an Amicon Ultra ultrafiltration unit with 10kDa cutoff (Millipore, Billerica, MA, USA) and exchange buffer (7 M urea, 2 M thiourea, 4 % [w/v] CHAPS, 30 mM Tris, pH 8.5). The protein amounts were determined in duplicate with a Bradford-based assay according to the manufacturer`s instructions (Roti®-Nanoquant, Carl Roth, Karlsruhe, Germany) with exchange buffer as control and aliquots stored at -20ºC. Protein labelling was performed with the “CyDye DIGE Fluor labeling kit” (GE Healthcare, Piscataway, NJ, USA) with “minimal DIGE” for healthy mice (50 µg/ 400pmol dye) and “saturation DIGE” for diabetic mice (5µg/ 0.25nmol dye) according to the manufacturer`s protocol. Immobilized pH gradient strips (pH 4-7, 24 cm; GE Healthcare) were incubated overnight in 650 μl rehydration buffer (7 M urea, 2 M thiourea, 4% [w/v] CHAPS, 20 mM [w/v] DTT, 0.5% [v/v] carrier ampholytes 3-10, Complete Mini protease inhibitor cocktail). Isoelectric focusing (IEF) after anodic sample cup-loading was carried out with the IPGphor3 system (GE Healthcare) under paraffin oil for 111.5 kVh. SDS-PAGE was performed overnight in polyacrylamide gels (12.5%) with the Ettan DALT II system (GE Healthcare) at 1-2 W per gel and 12ºC. Fluorescence signals were detected with a Typhoon 9400 (GE Healthcare). 2-D gels were analyzed with Delta2D 4.6 (Decodon, Greifswald, Germany).

**Mass spectrometry (MS) – Murine Skin and HaCat cell line**

For protein identification, additional 2-D gels were run with higher amount of unlabelled protein (400-800 μg) combined with 5 μg Cy3-labeled internal standard. After detection of the fluorescence signals (see above) and silver staining, labelled and unlabelled protein patterns were matched with the 2-DE image analysis software Melanie 3.09 (GeneBio, Geneva, Switzerland). Spots with correctly matched centers were excised, digested with trypsin (recombinant; Roche) and prepared as described by Ohlmeier *et al*., 2010 ^2^. The extracted and dried peptides were dissolved in 5 μl α-Cyano-3-hydroxycinnamic acid (98%, recrystallized from ethanol-water, 5 mg/ ml in 50 % [v/v] acetonitrile and 0.1 % [v/v] TFA) and 0.5 μl applied onto the sample plate using the dried-droplet method. Peptide masses were measured with an UltrafleXtreme MALDI-TOF/TOF (Bruker, Billerica, MA, USA). Proteins were identified according to their spot-specific peptide mass fingerprint and peptide sequences with the bioinformatic tool BioTools Version 3.2 (Bruker) and following search parameters (MS tolerance: 100 ppm, MSMS tolerance: 0.7 Da, enzyme: Trypsin, engine: Mascot version 2.4.0., database: NCBInr, modifications: Carbamidomethyl (Cys) and optional oxidation of Met, up to 1 missed cleavage).

**Western blotting using human skin and HaCat cells**

Total proteins from human skin and HaCaT cells were extracted using RIPA buffer (Cell Signaling Technology) supplemented with protease (Roche) and phosphatase inhibitor cocktail (Sigma-Aldrich). Skin samples were further homogenized with a TissueLyser LT (Qiagen) in the presence of beads (Qiagen, 69997). Protein concentrations in the supernatant were determined using Pierce BCA Protein Assay Kit (Thermo Fisher). Equal amounts of protein were resolved by SDS-PAGE and transferred electrophoretically onto nitrocellulose membranes (Bio-Rad, 741280). Membranes were incubated overnight at 4 °C with primary antibodies against the proteins of interest (Supplementary Table 5). After washing, membranes were incubated for 1 hour with a peroxidase-conjugated secondary antibody (Dako, polyclonal). Protein bands were visualized using 20× LumiGLO® Reagent and 20× Peroxide (Cell Signaling Technology, 7003). supplemented with protease (Roche) and phosphatase inhibitor cocktail (Sigma-Aldrich). For quantification, band intensities were measured using Image Lab software (version 6.1), with β-actin used as a loading control to normalize protein expression levels.

**Human Study design**

The controlled study was approved by the Northern Ostrobothnia Hospital District Ethics Committee (EETMK:5/2022). Participants were recruited through a randomized population study. Inclusion criteria for healthy and pre-T2D participants were: age 18–65 years, BMI 15–35 kg/m^2^, and fasting blood glucose of 3.9–5.6 mmol/L for healthy individuals or 6.0–6.4 mmol/L for pre-T2D individuals. Exclusion criteria included a history of cardiovascular disease, blood pressure >160/100 mmHg, or use of medications affecting blood pressure. Eligible volunteers attended two study visits. At the first visit, participants provided written informed consent, and baseline measurements including weight, height, body fat percentage, fasting blood glucose, and arterial blood pressure were recorded. A total of 31 participants were enrolled, comprising 14 pre-T2D and 17 healthy individuals.

The experimental protocol is illustrated in Supplementary Figure 1E. Following a 12-hour fast, fasting blood glucose levels were measured during the initial 45 minutes. Subsequently, skin biopsies, skin epidermal samples obtained via skin tape, and sweat samples were collected while participants remained at rest in a room temperature environment. Each participant consumed a 75g oral glucose solution (Suomen Diabeteskauppa Oy, Diabet Ab, LEMU, Finland) over a period of 5 minutes. Sweat collection began 5 minutes after glucose intake, with additional samples collected for a total of 45 minutes. Skin tape and skin biopsies were then collected. The endpoint of the experiment was set at 90 minutes from the start.

The epidermal layer was collected using a D-Squame standard sampling disc (D-100) along with a D-Squame pressure instrument (D-500, Clinical & Derm, USA). Following the manufacturer’s protocol, the wrist was first cleaned with 70% ethanol. The sampling disc was then applied, and pressure was exerted for 10 seconds using the D-Squame pressure instrument. A total of five sequential discs were collected from the same site.

Skin biopsies were obtained using a 3-5 mm punch from the abdominal region under local anesthesia with 1% lidocaine, with the addition of epinephrine when appropriate, following standard surgical procedures.

Sweat was collected using the Macroduct® Advanced Sweat Collection System (model 3710 SYS) via iontophoresis with 5% pilocarpine in water at 1.5 mA DC for 5 minutes.

**Isolation of RNA from epidermal layer and HaCat cells**

Total RNA was extracted from skin tape samples using Trizol reagent (Invitrogen, 15596-026) followed by purification with the RNeasy Mini Kit (Qiagen). For HaCaT cells, RNA was extracted directly using the RNeasy Mini Kit according to the manufacturer’s instructions.

Skin tape samples were incubated with Trizol for 5 min at 4°C and homogenized using TissuLyser^TM^ (Qiagen) for 5 min at 50 Hz. The homogenates were centrifuged at 13,000 rpm for 15 min at 4°C, and chloroform was added to the supernatants. Samples were incubated for 5 min at room temperature and centrifuged at 13000 rpm for 18 min at 4°C. The aqueous phase was collected, and the remaining extraction and purification steps were performed using the RNeasy Mini Kit. RNA quality from both skin and cell samples was assessed using a nanodrop spectrophotometer.

To analyse the mRNA expression level of *DLST*, 2μg of RNA was reverse transcribed to cDNA using the SuperScript™ VILO™ cDNA Synthesis Kit (ThermoFisher, 11754050) or the First Strand cDNA synthesis kit (Roche Applied Science). RT-qPCR and qPCR were performed for *DLST*, *18S* and *GAPDH* using TaqMan gene expression assays or the SYBER Green method. Primer sequences used are listed in Supplementary Table 6. PCR reactions were run for 40 cycles following initial polymerase activation (50 °C for 2 min) and initial denaturation (95 °C for 20 min), with subsequent cycling conditions of 95 °C for 1 s (denaturation) and 60°C for 20s (annealing/extension). Relative target gene expression was normalized to *18S* or *GAPDH* expression, and fold changes were calculated using the ΔΔCT method, followed by statistical analysis with a two-tailed Student’s t-test.

**HaCat cell culture and glucose treatment**

Human keratinocytes cell line (HaCat) was kindly provided by Dr. Rezvani HR (Inserm, BMGIC, UMR 1035, Bordeaux, France). Cells were cultured in DMEM (11885084, Thermo Fisher) supplemented with 1% Penicillin/Streptomycin (Sigma), 10% FBS (Gibco) and maintained at 37°C in a humidified incubator under 5% CO2. To establish a high-glucose environment mimicking diabetic conditions, cells were transferred to high-glucose DMEM (11965092, ThermoFisher) for three consecutive passages before use in the experiments described in this study.

**Primary keratinocytes culture and glucose treatment**

Primary keratinocytes were isolated from the skin of 6-8-week-old mice as previously described (^1^). Briefly, mice (C57/BL6) were sacrificed (by cervical dislocation) and the hair shaved from their backs. The skin was then sterilized by immersion in a beaker of 10% betadine for 2 min, in a beaker of 70% ethanol for 1 min and then in sterile Dulbecco’s phosphate (PBS) for 1 min. The skin samples were treated with trypsin for 2h at 33°C to separate the epidermis from the dermis. Keratinocytes were cultured in F12 (21765-037) – DMEM (41965-062), medium, both from Thermo Fisher, supplemented with hydrocortisone (0.5 µg/ml), epidermal growth factor (EGF) and insulin (5 µg/ml), all from Sigma. The medium was changed three times a week. When the cultures reached 70–80% confluence the keratinocytes were incubated with two concentrations of glucose (G7021-Sigma) (5 mM or 25 mM) for 45 min and then collected for subsequent experiments.

**Generation of DLST knockdown HaCat cell line**

To generate DLST KD cell line, high-glucose adapted HaCaT cells were transduced with short hairpin RNA (shRNA) targeting human DLST (TRCN0000344818, Sigma-Aldrich) or a scrambled shRNA control (shCTRL) cloned into a pLKO.1-puro-CMV-tGFP custom vector (Sigma-Aldrich). Transduced cells were selected using flow cytometry to determine the percentage of GFP-positive cells, followed by puromycin selection. Non-transduced (NT) cells were used as a negative control to assess whether the control vector affected cell proliferation or induced other molecular changes, as described in the Results section. The three cell lines; NT, shCTRL, and shDLST; were used to assess the efficiency of DLST knockdown. For metabolomics and proteomics analyses, only the shCTRL and shDLST cell lines were included. Data were obtained from three independent experiments corresponding to cell lines (Exp 1–3), as described in the main manuscript, and were combined for analysis. In contrast, Exp. 4 is presented as a single, separate analysis.

**α-KGD activity assay**

HaCaT cells, including NT, shCTRL, and shDLST lines, were detached using Trypsin-EDTA, collected, and the resulting cell pellets were incubated in 200 µl of assay buffer (50 mM potassium phosphate, pH 7.4, containing 0.05% Triton X-100 [Sigma] and 0.5 mM dithiothreitol [DTT; Acros Organics]) to achieve cellular permeabilization ^3–5^. The α-KDH activity assay was performed in the assay buffer containing 5% cell suspension, 0.1 mM CoA, and 0.1 mM NAD⁺, using a UV–Vis dual-beam spectrophotometer (Shimadzu) with a wavelength pair of 340 nm and 385 nm at 22 ^o^C, as previously described ^3^. After recording the background signal, the reaction was initiated by adding 0.8 mM α-KG. The α-KDH activity was calculated by using an extinction coefficient (ε) of 5.33 x 10^3^ L·mol^-1^·cm^-1^, as reported previously ^6^. Data were normalized to total protein concentrations using NanoDrop™ 2000/2000c spectrophotometer (Thermo Fisher Scientific), following the provider instructions.

**Chromatin immunoprecipitation (ChIP) assay**

HaCat cells from both shCTRL and shDLST groups, maintained in high-glucose conditions, were fixed with 1% formaldehyde and cell pellets were collected. For chromatin shearing, pellets were sonicated at 4°C for 30s on /59s off, repeated 10 times. Soluble chromatin was obtained by centrifugation at 16,000 x g for 10 min at 4°C. DNA was extracted using GeneJET PCR Purification Kit (ThermoFisher) in accordance with the manufacturer’s instructions, and fragment size (̴ 250 bp) was verified by 1.5% agarose gel.

Protein–DNA complexes were immunoprecipitated with 6 µg of anti-Sp1 antibody, with normal IgG used as a negative control. Immunoprecipitated DNA was purified, eluted in 50 µL of elution buffer, and stored at −20 °C until analysis. RT-qPCR was performed on eluted DNA using SYBR Green and primer sets listed in Supplementary Table 7, following the manufacturer’s protocol. Thermocycling conditions were: 95 °C for 10 min, followed by 40 cycles of 95 °C for 15 s, 60 °C for 30 s, and 72 °C for 30 s. The relative enrichment of target gene promoter regions was calculated using Ct values and reaction efficiencies, with normalization to IgG control levels.

**Sp1 overexpression in HaCat cell line**

To generate Sp1-overexpressing (Sp1 OE), HaCaT keratinocytes, cells were transfected with the full-length human Sp1 cDNA expression plasmid, as previously described ^7^. The pcDNA3-Flag-SP1 plasmid (Addgene plasmid #232649), originally deposited by Dr. Chen-Yang Shen, was obtained from Addgene. HaCaT keratinocytes were seeded at a density of 2.5 × 10⁶ cells per condition and transfected with the Sp1 expression plasmid using FuGENE® 6 transfection reagent 1:3 ratio (Promega, E2691) according to the manufacturer's instructions. Cells transfected with the corresponding empty vector plasmid (pcDNA3; vehicle) were used as controls. 48hours, following transfection, cells were collected and the Sp1 overexpression was confirmed by qPCR. Additionally, DLST expression level was examined. GAPDH and tubulin were used as references genes.

**Sp1 inhibition in HaCat cell line**

To inhibit Sp1 activity in keratinocytes, HaCaT keratinocytes were seeded at a density of 2.5 × 10⁶ cells per condition and treated with the Sp1 inhibitor mithramycin (M6891, Merck Life Science) or DMSO as a vehicle control (final concentration 0.1%) in culture medium. After 24 h of incubation with different concentrations of mithramycin (100 nM, 250 nM, 500 nM, and 1 µM) or DMSO, cells were collected for qPCR analysis. The expression levels of SP1, DLST, and HIF-1α were evaluated, with GAPDH used as the reference gene for normalization.

**Targeted LC-MS metabolomics analysis – HaCat cell line, human skin, and sweat**

For Targeted metabolomics profiling, the metabolites were extracted from cells using 400µl of cold LC-MS grade extraction solvent (Acetonitrile:Methanol:MQ; 40:40:20) and subsequently, samples were vortexed for 2 min and sonicated for 1 min followed by centrifugation at 14000rpm at 4°C for 5 minutes. For tracer analysis, cells were treated with xmM U13C-Glucose in MQ or 2mM U13C-Glutamine (Cambridge Isotope laboratories) for 30 and 120min before extraction? After centrifugation supernatants were transferred into polypropylene tubes and placed into Nitrogen gas evaporator and dried samples were suspended with 40µl of extraction solvent (ACN:MeOH:MQ; 40:40:20) and vortexed for 2 min and transfered into HPLC glass auto sampler vials. 2µl of samples were injected with Thermo Vanquish UHPLC coupled with Q-Exactive Orbitrap quadrupole mass spectrometer equipped with a heated electrospray ionization (H-ESI) source probe (Thermo Fischer Scientific). A SeQuant ZIC-pHILIC (2.1×100 mm, 5-μm particle) column (Merck) was used for chromatographic separation. The gradient elution was carried out with a flow rate of 0.100 ml/minutes with using 20mM ammonium hydrogen carbonate, adjusted to pH 9.4 with ammonium solution (25%) as mobile phase A and acetonitrile as mobile phase B. The gradient elution was initiated from 20% Mobile phase A and 80% of mobile phase B and maintain till 2min., followed by 20% Mobile phase A gradually increasing up to 80% till 17 min., then 80% to 20% Mobile phase A decrease in 17.1 min. and maintained up to 24 minutes. The column oven and auto-sampler temperatures were set to 40 ± 3 °C and 5 ± 3 °C, respectively. MS was equipped with a heated electrospray ionization (HESI) source using polarity switching and following setting: resolution of 35,000, the spray voltages: 4250 V for positive and 3250 V for negative mode, the sheath gas: 25 arbitrary units (AU), and the auxiliary gas: 15 AU, sweep gas flow 0, Capillary temperature: 275°C, S-lens RF level: 50.0. Instrument control operated with the Xcalibur 4.1.31.9 software (Thermo Fischer Scientific). The peak integration was done with the TraceFinder 4.1 software (Thermo Fischer Scientific) using confirmed retention times for 450 metabolites standardized with library kit MSMLS-1EA (Merck). Peak smoothening was adjusted to 7 and sample peak threshold set to 50 000 m/z. The data quality was monitored throughout the run using pooled QC sample prepared by pooling 5µl from each suspended samples and interspersed throughout the run as every 10th sample. The metabolite data was checked for peak quality (poor chromatograph), % RSD (20% cutoff) and carryover (20% cutoff).

**Metabolomics data analysis**

Identified metabolites were normalized to cell number prior to analyzing data using Metaboanalyst 5.0 (www.metaboanalyst.ca).

Missing values were replaced by the limit of detection, set as 20 % of the minimum positive value of each metabolite and normalization of all samples was made by using log transformation. Metabolites with a fold change of 1.5 and a p-value of 0.1 using unpaired non-parametric t-test were considered significant.

**Statistical analysis**

All data analyses were performed using GraphPad Prism software (version 9.3.1). A two-tailed Student’s t-test was applied, and P-values less than 0.05 were considered statistically significant.

**Supplementary Figures**

**Supplementary Figure 1. Identification of DLST as a glucose-responsive protein in human and mouse skin. A.** Representative 2D-gel of skin proteins (5μg) from the skin of PBS-injected healthy mice. **B.** Western blot analysis of DLST in the skin of healthy and T1D mice injected with either PBS (Ctrl)- and glucose-injected (2g/kg, T) healthy and T1D mice. Skin biopsies were collected at 45 min post-injection from four groups (n=6/group) and subjected to western blotting analysis using specific anti-DLST antibody, β-actin was used as loading control. **C.** Quantification of DLST protein expression levels in healthy and T1D mice. Results are presented as means fold change after normalization to control-injected mice (n=6/group for healthy, n=4/group for T1D). **D.** Quantification of DLST protein levels at fasting in healthy and T1D mice using LC-MS spectrometry. **E.** RT-qPCR analysis of *Dlst* mRNA levels in murine primary keratinocytes treated with 5 mM or 25 mM for 45 min. Data are shown as mean ± SEM, normalized to control values, from four different mice, with each measurement performed in duplicate. **F.** Schematic diagram illustrating the clinical pilot study workflow. **G.** qPCR analysis of *DLST* mRNA levels in human epidermal samples collected from healthy and pre-T2D individuals at fasting. Data are shown as mean ± SEM, normalized to control values, from three different individuals, with each measurement performed in triplicate. Statistical analysis was performed using Two-tailed Student’s t-test. *P* ≤ 0.05 was considered statistically significant.

**Supplementary Figure 2. DLST Knockdown reveals a role in metabolic reprogramming under high-glucose conditions in keratinocytes. A-H.** Targeted metabolomics analysis demonstrated substantial alterations in multiple metabolic pathways in shDLST versus shCTRL cells cultured under high glucose conditions, including TCA cycle (**A**), purine and pyrimidine metabolism (**B-D**), redox cofactors (**E**), creatine biosynthesis (**F**), fatty acid metabolism (**G**), and amino acids and derivatives (**H**). Data are presented as fold changes in peak area ± SEM of three independent experiments after normalization to the control. **I.** A graphical summary illustrates the metabolic pathways influenced by DLST silencing under hyperglycemic conditions. Statistical comparisons were conducted using a two-tailed Student’s t-test, with *P* ≤ 0.05 considered statistically significant. α-KG: alpha ketoglutarate. AMP: Adenosine monophosphate. ADP: Adenosine diphosphate. ATP: Adenosine triphosphate. GMP: Guanosine diphosphate. GDP: Guanosine diphosphate. IMP: Inosine monophosphate. UMP: Uridine monophosphate. UDP: Uridine diphosphate. UTP: Uridine triphosphate. CMP: Cytidine monophosphate. NAD/NADH: Nicotinamide adenine dinucleotide/ Nicotinamide adenine dinucleotide (reduced form). NADP/NADPH: Nicotinamide adenine dinucleotide phosphate/ Nicotinamide adenine dinucleotide phosphate (reduced form).

**Supplementary Figure 3. Blood glucose levels influence metabolic profiles in healthy human skin.** Panels **A-C** illustrate changes in skin metabolites from healthy individuals (n=8) observed 45 minutes after oral glucose ingestion, under conditions of elevated blood glucose levels. An increasing trend was noted in the levels of glutamate (**A**), glutamine (**B**), and carnitine (**C**). Data are presented as fold changes in peak area ± SEM, based on eight independent participants, normalized to baseline (control) values. Statistical comparisons were conducted using a two-tailed Student’s t-test, with *P* ≤ 0.05 considered statistically significant.

**Supplementary Figure 4. Blood glucose levels influence metabolic profiles in pre-T2D human skin.** Panels **A-J** illustrate changes in skin metabolites from individuals with pre-T2D (n=3) observed 45 minutes after oral glucose ingestion, under conditions of elevated blood glucose levels. A decreasing trend was noted in the levels of 2-HB (**A**), glutamate (**B**), lactate (**C**), alanine (**D**), Lysine (**E**), threonine (**F**), tyrosine (**G**), pyruvate (**H**), carnitine **(I)**, and Creatine (**J**). Data are presented as fold changes in peak area ± SEM, normalized to baseline (control) values for each participant. Statistical comparisons were conducted using a two-tailed Student’s t-test, with *P* ≤ 0.05 considered statistically significant.

**Supplementary Figure 5. DLST expression in the skin of healthy mice.** Uncropped blot showing DLST expression levels in the skin of healthy mice injected with either PBS (indicated as “Ctrl= control”, n=6) or D-glucose (2g/Kg, indicated as “T= Treated”, n=6), 45 min post-injection. The DLST protein band was detected at a molecular weight of approximately 49 kDa and was used for quantification after normalization to β-actin expression levels.

**Supplementary Figure 6. DLST expression in the skin of T1D mice.** Uncropped blot showing DLST expression levels in the skin of T1D mice injected with either PBS (indicated as “Ctrl= control”, n=4) or D-glucose (2g/Kg, indicated as “T= Treated”, n=4), 45 post-injection. The DLST protein band was detected at a molecular weight of approximately 49 kDa and was used for quantification after normalization to β-actin expression levels.

**Supplementary Figure 7. DLST expression levels in HaCat cell line.** Uncropped blot of DLST expression levels in HaCat keratinocytes cultured in 5mM and 25 mM glucose conditions for at least three passages. The DLST protein band was detected at a molecular weight of approximately 49 kDa and was used for quantification after normalization to β-actin expression levels.

**Supplementary Figure 8. E1 (OGDH) and E3 (DLD) expression levels in HaCat cell line.** **A.** Western blot analysis of E1 and E3 subunits in HaCaT keratinocytes cultured in high-glucose conditions (25 mM) for at least three passages versus those maintained in physiological glucose levels (5 mM), using four independent experiments, β-actin was used as loading control. **B.** Quantification of E1 and E3 band intensities in HaCaT cells shows no changes in protein expression in response to chronic high-glucose exposure. Data represent mean fold change ± SEM.

**Supplementary Figure 9. E1 protein expression in HaCat cell line.** Uncropped blot of E1 expression levels in HaCat keratinocytes cultured under 5mM and 25 mM glucose conditions for at least three passages. The E1 band was detected at a molecular weight of approximately 115 kDa and was used for quantification after normalization to β-actin expression levels.

**Supplementary Figure 10. E3 protein expression in HaCat cell line.** Uncropped blot of E3 expression levels in HaCat keratinocytes cultured under 5mM and 25 mM glucose conditions for at least three passages. The E3 band was detected at a molecular weight of approximately 54 kDa and was used for quantification after normalization to β-actin expression levels.

**Supplementary Figure 11. Sp1 modulation influences HIF-1α transcription level. A.** *HIF-1α* mRNA expression levels were quantified by qPCR following Sp1 overexpression (Sp1 OE) in HaCat cell line. **B.** *HIF-1α* mRNA expression levels were quantified by qPCR following pharmacological inhibition of Sp1 activity with mithramycin in HaCaT cells. In both panels, each condition included three biological replicates, and all samples were analyzed in technical triplicate. NT, non-transduced cells; Vehicle, cells transduced with the empty pcDNA3 vector; Sp1 OE, cells transduced with the full-length human Sp1 cDNA expression plasmid (pcDNA3-Flag-Sp1). DMSO, dimethyl sulfoxide, used as the vehicle control; Mith, mithramycin, used as a specific inhibitor of Sp1 activity; nM, nanomolar; µM, micromolar. Statistical analysis was performed using Two-tailed Student’s t-test. *P* ≤ 0.05 was considered statistically significant.

**Supplementary Table 1. Details about the proteomic changes in the skin of healthy (H) and type 1 diabetic mice (T1D) mice.** Changed spots. corresponding proteins in control (Co) and glucose-treated (G) mice, along with its descriptions and UniProt-accession numbers. The ratio reflects the change in mean normalized spot volumes, and statistical significance is indicated using the t-test (*P* ≤ 0.05).

**Supplementary Table 2. Details about the protein identification.** MS and MSMS Mascot scores, sequence coverage (SC), and the covered peptides (P) are shown. The total number of amino acids (aa) and the sequence covered by MS are also indicated. If the identified spot allowed for the presence of several isoforms, amino acids and sequence coverage are reported for the most common isoform.

**Supplementary Table 3. Characteristics of participants in this study.** Data are presented as means ± SD. Participants were divided into two groups: Healthy controls (n= 17) and pre-T2D (n=14). T2D= Type 2 Diabetes. BMI= Body Mass Index. BG. Blood glucose. F= Female. M= Male.

**Supplementary Table 4. Metabolic profiles of pre-T2D skin versus healthy skin.** The table illustrates the fold changes of 40 metabolites in pre-T2D skin (n=3) compared to healthy skin (n=3). The majority of metabolites showed a trend toward increased levels in pre-T2D skin, with ornithine being the only metabolite reaching statistical significance. In contrast, aconitate, E4P, and linoleate exhibited a trend toward decreased levels. Data are presented as fold changes in peak area ± SEM, normalized to control values in healthy skin samples. Statistical comparisons were performed using a two-tailed Student’s t-test, with *P* ≤ 0.05 considered statistically significant.

**Supplementary Table 5. List of antibodies used in this study.** DLST= dihydrolipoyl-succinyltransferase. Sp1= Specificity protein. IgG= Immunoglobulin G. β-actin= beta actin.

**Supplementary Table 6. List of primer sequences used for quantitative RT-qPCR in this study.** The primers corresponding to *Sp1* and *tubulin* were purchased from Sigma-Aldrich, and their sequences are provided below. Primers that target *Dlst*, *18S* and *GAPDH* were purchased from ThermoFisher as TaqMan assays, with their references number indicated. GAPDH= Glyceraldehyde-3-phosphate dehydrogenase.

**Supplementary Table 7. List of primer sequences used for quantitative ChIP RT-qPCR in this study.**
